# Comparison between European Medicines Agency and US Food and Drug Administration in Granting Accelerated Marketing Authorizations for Covid-19 Medicines and their Utilized Regulations

**DOI:** 10.1007/s43441-023-00574-6

**Published:** 2023-10-20

**Authors:** Marina Ghadanian, Ellen Schafheutle

**Affiliations:** Division of Pharmacy & Optometry, Faculty of Biology, Medicine & Health, School of Health Sciences, The University of Manchester, Manchester, UK

**Keywords:** USFDA, EMA, Covid-19, Regulations, Conditional, Emergency use, Marketing authorizations

## Abstract

**Background:**

Prompted by the Covid-19 pandemic and the need to ensure timely and safe access to medicines during a pandemic, the aim of this study was to compare and contrast the EU and US regulations, processes, and outcomes pertaining to the granting of accelerated Marketing Authorizations (MAs) for COVID-19 vaccines and treatments with a view to determining how effective these regulations were in delivering safe medicines in a timely manner.

**Methods:**

MAs for medicines approved for Covid-related indications in the first two pandemic years (March 2020–February 2022) were identified using the European Medicines Agency (EMA) and US Food and Drug Administration (FDA) websites. Authorization reports and utilized regulations were reviewed to determine and compare approval timelines, facilitated pathways, accepted clinical evidence, and effectiveness of the regulations by assessing them against time and safety standards.

**Results:**

By the end of February 2022, the EMA and FDA had granted 12 and 14 MAs, respectively. Two EU and two US approvals were issued in relation to new indications for already-approved treatments; the remaining ones were first-time approvals of novel vaccines and treatments. The median time to approval was 24 days for the EMA’s conditional MAs and 36 days for the USFDA’s Emergency Use Authorizations (EUA) for all Covid-19 medicines. This is compared with 23 and 28 days, respectively, specifically for first-time novel vaccines and treatments authorized by both USFDA and EMA. The USFDA and EMA differed markedly in terms of the time taken to approve new indications of already-approved treatment; the USFDA took 65 days for such approval, compared with 133 days for the EMA. Where MAs were issued by both authorities, USFDA approvals were issued before EMA approvals; applications for approval were submitted to the FDA before submission to the EMA. Three EU and two US MAs were based on data from two or more phase 3 clinical trials; the remaining ones were based on single trial data. Only six EU and four US trials had been completed by the time of authorization. This was in line with regulations. While the applicable regulations shared many similarities, there were marked differences. For instance, the EU’s conditional MA regulation pertains only to first approvals of new treatments. It does not cover new indications of already-approved treatments. This contrasts with the US, where the EUA regulation applies to both types of applications, something that may have impacted approval timelines.

Overall, both EU and US utilized regulations were considered to be effective. For most cases, utilizing such regulations for Covid-19 MAs resulted in faster approval timelines compared to standard MAs. They were flexible enough to manage the process of granting emergency approvals while maintaining strict requirements and allowing comprehensive reviews of the supporting evidence.

**Conclusion:**

US and EU regulations were effective in ensuring timely accelerated market access to Covid-19 medicines during the pandemic without compromising the approval standards related to safety or efficacy. The population in both regions will receive comparable access to medicines during a pandemic if sponsors submit their applications to both authorities in parallel.

**Supplementary Information:**

The online version contains supplementary material available at 10.1007/s43441-023-00574-6.

## Introduction

The World Health Organization (WHO) declared Covid-19 a global pandemic in March 2020 [1]. Consequently, many pharmaceutical companies and sponsors initiated clinical trial programs to find safe and effective vaccines and/or treatments. These trials were either to support authorization of new molecular entities or for approval of additional therapeutic indications of already-authorized medicines. By 2021, some vaccines and treatments had received emergency use or conditional marketing authorization (MA) both in the European Union (EU) and United States of America (USA) on an accelerated basis [2–4].

Market access of new medicines is highly regulated in most developed countries worldwide. The regulatory authorities are responsible for reviewing the safety, efficacy and quality evidence provided by pharmaceutical companies in order to grant marketing authorizations (MAs) for new medicines or for new therapeutic indications of already-authorized medicines. The US Food and Drug Administration (USFDA) and European Medicines Agency (EMA) are the regulatory authorities in the USA and EU, respectively, and considered the biggest regulatory authorities worldwide.

Both authorities have their own authorization procedures, requirements, and MA application review timelines. For standard new innovative drugs, the timelines from development to final approval can be lengthy and reach 10–20 years [5]. However, both authorities have facilitated pathways and tools to accelerate the development and approval timelines for medicines which fulfill certain criteria, such as if the medicine is for life-threatening disease where there are no alternatives, if it provides substantial improvement over existing treatments, or if it is for public health threats [6–12]. There are different types of facilitated pathways in the USA and EU that have been introduced gradually over the past decades, each with set eligibility criteria and conditions. There were reports that the EMA was more restrictive than USFDA in granting facilitated designations for drugs not having high therapeutic value between 2007 and 2017; i.e., many drugs, which were rated as having low therapeutic value, received expedited approvals by USFDA but not by EMA [13, 14].

In addition, although sponsors may submit the same clinical evidence for the same indications and other details about the use of a medicine, the regulatory authorities may not approve them as they are and request some changes. For instance, differences have been found between the indications of new medicines in USA and EU [15, 16], as well as routes of administration, dosage forms, strengths, and posology of priority review products approved by USFDA and EMA. Between 1999 and 2011, the EMA approved more indications than USFDA for the same drugs, yet EMA-approved indications were more restrictive, i.e., the drug indication or use was limited to factors such as the disease state, previous therapy failure, or inappropriate alternative therapies [15].

The Covid-19 pandemic presented an unprecedented global threat, which made fast approvals of effective and safe treatments paramount. Indeed, regulatory authorities globally granted emergency or conditional approvals to Covid-19 vaccines and treatments, including by the USFDA and EMA. However, approvals via facilitated pathways differ between the USFDA and EMA, and it was not known if the population of both regions had comparable access to officially authorized lifesaving medicines. Secondly, the speed of approvals led to concerns over potentially compromising the safety and efficacy standards of Covid-19 medicines [17–19]. Such concerns had previously been noted in relation to pre-Covid-19 use of facilitated approval pathways, including drug safety concerns, insufficient or delayed studies to confirm preliminary evidence, potential regulatory capture associated with the fees paid for expedited review, and public misperception of the therapeutic value of drugs approved via expedited pathways [20].

## Background on Marketing Authorizations of New Medicines

This section provides an overview of regulatory pathways for medicines MAs, in order to describe and compare the different application pathways in USA and EU.

### Regulatory Pathways Overview in USA

After completing clinical trials, and in line with the standard new drug application approval pathway described in Federal Food, Drug, and Cosmetic Act 505(b), a New Drug Application (NDA) is submitted containing full safety and efficacy reports to USFDA’s Center for Drugs Evaluation and Research (CDER) for all new innovative drugs to be approved for market entry [21]. Section 505(j) of the same Act specifies that the approval of all generic drug products is via an Abbreviated New Drug Application (ANDA) pathway, with evidence that the generic drug is equally safe and effective as the approved drug.

Once the application is filed, the Code of Federal Regulations stipulates that the FDA review should be completed within the specified timeframe of 180 days, which is subject to extension if required [22]. Following this, the FDA issues either an approval letter to market if there are no refusing reasons, or a response letter detailing deficiencies and recommendations to make the application viable [22].

The licensing of biological products is regulated under separate regulation section. As per the Public Health Service Act, a Biological License Application (BLA) is submitted to the USFDA’s Center for Biologics Evaluation and Research (CBER) or (CDER) instead of NDA. The BLA should contain data derived from non-clinical laboratory and clinical studies which demonstrate that the manufactured product meets prescribed requirements of safety, purity, and potency. USFDA similarly issues complete response letter detailing deficiencies and recommendations if any [23].

In practice, NDAs and BLAs have 10-month standard review timeline or 6-month priority review timeline; both following a 60-day filing period. This is to meet the performance goals for the review timelines set by the Prescription Drug Used Fee Act (PDUFA), which was first created by Congress in 1992 to authorize the FDA to collect fees from companies to expedite the drug approval process [113].

The USFDA has the following regulatory pathways to expedite the development and application review timelines of both drugs and biologics in cases of unmet medical need or for serious life-threatening conditions, [6]:

- Fast track designation
- Breakthrough therapy designation
- Accelerated approval
- Priority review

Table 1 summarizes the details extracted from the USFDA’s guidance for expedited programs [6].

**Table 1:** USFDA expedited programs for new drugs approvals

Expedited program	Fast track designation	Breakthrough therapy designation	Accelerated approval	Priority review
**Eligibility criteria**	Drug is for serious condition AND pre-clinical or clinical data demonstrate the potential to address unmet need OR The drug is designated as a qualified infectious disease product	Drug is for serious condition AND its preliminary clinical evidence indicates substantial improvement on a clinically significant endpoint(s) over available therapies	Drug is for serious condition AND has advantage over available therapies AND Demonstrates an effect on a surrogate endpoint that predicts clinical benefit or clinical endpoint that can be measured earlier than irreversible morbidity or mortality	Drug is for serious condition AND Provides significant improvement in safety or effectiveness OR The drug is designated as a qualified infectious disease product OR Any supplement for labeling change to a report on a pediatric study under 505Ab OR Any application or supplement submitted with a priority review voucher
**Requesting time**	With or after IND No later than pre-NDA or pre-BLA meeting	With or after IND No later than end of phase 2 meeting	Possibility of accelerated approval is discussed with review division during development	With original NDA, BLA, or efficacy supplement
**Expediting features**	Actions to expedite development and approval Rolling review*	Actions to expedite review Rolling review* Guidance on efficient development Organizational commitment	Approval based on surrogate endpoint or intermediate clinical endpoint likely to predict a drug’s clinical benefit	Shorter review of Marketing application (6 months compared with the 10-month standard review as per PDUFA goals)

^*^Rolling Review means that a drug company can submit completed sections of its BLA or NDA for review by FDA, rather than waiting until every section is completed before the entire application can be reviewed [56]

These facilitated pathways have been successfully used since 1987 [20, 24–27]. Nearly half of the approvals via facilitated pathways were for first in class medicines [20, 24], and the most common disease class was oncology treatments [25]. “Priority review” has been used the most, and “accelerated approval” the least [24, 25], with the combination of facilitated pathways having been shown to be more efficient in shortening the development and review times compared to “priority review” alone [26]. In addition to these expedited programs, the Emergency Use Authorization (EUA) legal tool allows the USFDA to protect the public against chemical, biological, radiological, and nuclear threats including infectious diseases by facilitating the availability and use of medical countermeasures needed during public health emergencies [7, 28]. Medical countermeasures include biologics such as vaccines, drugs, and medical devices [29]. In 2009, USFDA issued EUAs during the influenza H_1_N_1_ pandemic for many antiviral drugs such as Oseltamivir. In 2011, they issued EUAs for doxycycline for inhalational anthrax post-exposure prophylaxis. EUA was used successfully during anthrax and influenza H_1_N_1_ pandemics and they were considered first major tests for this legal tool [27].

### Regulatory Pathways Overview in European Union

In the European Union, Directive 2001/83/EC [30] allows several procedures for marketing authorization of new medicines:

- **Centralized Procedure: “Marketing Authorization Application”** is submitted to EMA, and following approval, the European Commission issues final authorization, valid for all EU member states.
- **Decentralized Procedure:** The same MA application is submitted simultaneously to several EU member states. The determined Reference Member State leads the assessment. Upon approval, all member states issue national authorizations.
- **Mutual Recognition Procedure:** If a medicine has a national authorization in one EU member state, the MA application can be submitted in other member states, which relies on the original evaluation.
- **National Procedure:** The MA application is submitted through the national procedure in one EU member state, if the medicine is intended only for that member state.

All medicinal products, whether biological or chemical, will go through the same type of MA application.

EMA MA follows the centralized procedure (EC 726/2004), which is the compulsory route for many new innovative medicines indicated for treatment of certain diseases such as cancer, diabetes, viral diseases, and acquired immune deficiency syndrome. It is also compulsory for orphan and advanced therapy medicinal products, and for medicinal products developed by certain biotechnological processes such as by recombinant DNA technology or hybridoma, and monoclonal antibody methods [31]. The EMA’s Committee for Medicinal Products for Human Use must give its opinion within 210 days of receipt of a valid application.

The following facilitated pathways are available under the EU centralized procedure:

- **Conditional MA:** Under EC No 507/2006 [32], a conditional MA can be granted without comprehensive safety and efficacy clinical data, but only if the medicinal product is for public health benefit which outweighs risks, if unmet medical need is fulfilled, and if the applicant can provide comprehensive data in future. This is applicable for medicinal products for seriously debilitating or life-threatening diseases; or for medicinal products needed in emergency situations, or for orphan designated medicinal products for rare diseases, or in response to public health threats duly recognized either by the WHO or by the Community in the framework of Decision No 2119/98/EC. The EMA has additional detailed guidelines for granting conditional MAs [8].
- **MA under Exceptional Circumstances:** Under Article 14(8) of Regulation (EC) No 726/2004 [31], the EMA can grant authorization under specific procedures in exceptional circumstances for objective verifiable reasons, specifically when the applicant can justify that comprehensive data cannot be provided. This authorization can only be continued with annual assessment of the conditions. The EMA has guidance for these specific procedures [9].
- **Accelerated Assessment:** Article 14 (9) of Regulation (EC) No 726/2004 [31] states that the applicant can request accelerated assessment for a MA application if the medicinal product has public health interest and if it demonstrates therapeutic innovation. If the request is accepted, the application review timeline can be reduced from 210 to 150 days. Further EMA guidance on this procedure exists [10].
- **Priority Medicines (PRIME) Scheme:** This was launched by EMA in 2016, also based on the accelerated assessment permitted under Article 14 (9) of Regulation (EC) No 726/2004. This scheme aims to support the development of a medicinal product if it is for unmet medical need or if it has advantage over other existing therapies, by enhancing the interaction and early dialogue with the developers and providing scientific advice [11, 12].

Table 2 summarizes the details of EMA facilitated pathways for new MAs.

**Table 2:** EMA facilitated pathways for new marketing authorizations

Facilitated pathway	Conditional MA	MA under exceptional circumstances	Accelerated assessment	Priority medicines (PRIME) scheme
**Eligibility criteria**	If comprehensive safety and efficacy clinical data are not provided, but if the applicant is able to provide comprehensive data in future For medicinal products for seriously debilitating or life-threatening diseases, or for medicinal products needed in emergency situations, or in response to public health threats duly recognized either by the World Health Organization or by the Community in the framework of Decision No 2119/98/EC; or for orphan designated medicinal products for rare diseases	If comprehensive data cannot be provided and the applicant can justify, authorization can be granted under specific procedures in exceptional circumstances for objective verifiable reasons	If the medicinal product has interest from a public health point of view and if it demonstrates therapeutic innovation	If the medicinal product has major public health interest from therapeutic innovation point of view and it is for unmet medical need or if it has advantage over other existing therapies
**Requesting time**	At the time of application for marketing authorization. *(It may be requested by the applicant or proposed by the CHMP)*	In advance of the Marketing Authorization application	2–3 months before the actual submission of the marketing authorization application in order to allow sufficient time for its assessment	Early stages of development; from exploratory clinical trial phase with proof of concept, or in exceptional cases at an earlier stage of development
**Expediting features**	Authorization based on the obligations to provide comprehensive data in future. However, it is valid for one year subject for renewal and assessment of specific obligations	Authorization without comprehensive data. However, it is subject to annual assessment of the conditions and justifications	Application review timeline is reduced to 150 days instead of the standard 210 days	Support provided for the development of the medicinal product by enhancing the interaction and early dialogue with the developers and providing scientific advice

Interestingly, between 1995 and 2016, the conditional MA pathway did not accelerate the approval process for innovative drugs; in fact, review timelines were longer than standard review timelines due to longer administrative periods between EMA’s final decision and the official European Commission decision [33, 34]. Furthermore, longitudinal analysis conducted on the implementation of the conditional MA instrument emphasized that the majority did not use the conditional MA pathway in a prospectively planned fashion but instead this option appeared to be used when submitted data were not strong enough to justify the standard approval [34]. Use of the exceptional circumstances pathway also did not accelerate the approval process between 2006 and 2009, due to longer clock-stop periods for the sponsor to respond to EMA’s queries [33].

### Similarities and Differences in Regulatory Pathways between USA and EU

In both regions, the related regulatory authorities (USFDA and EMA) require that an application is submitted for medicinal products for review and approval before market entry. In USA, there is one submission procedure with different types of applications, i.e., NDA for drugs or BLA for biologicals. In the EU, there is one type of MA application for drugs and biologicals, but under four different procedures, i.e., centralized, decentralized, mutual recognition, and national procedures.

USFDA and EMA have standard timelines to review applications. In both regions, facilitated pathways exist to accelerate development and/or the review timelines for exceptional cases such as medicinal product for life-threatening disease or without alternatives. However, the pathways, terminologies, and their conditions differ between the two regions. Some are equivalent pathways with different terminologies, such as the US breakthrough therapy designation and the EU priority medicines (PRIME) scheme. Other examples are the US fast track designation or priority review and the equivalent EU accelerated assessment, and the US emergency use authorization and EU conditional MA for emergency authorizations during recognized public health threats.

Some pathways are specific to each region with no equivalent pathway in the other region, such as the EU MA under exceptional circumstances, which approves without comprehensive clinical confirmatory studies under specific conditions.

Table 3 summarizes the previously mentioned applications and pathways available in USFDA and EMA centralized procedures to demonstrate the equivalency in both regions.

**Table 3:** USFDA and EMA equivalent applications and pathways

		US Food and Drug Administration	European Medicines Agency
**Marketing authorization approvals—standard applications**		New Drug Application (NDA) Biological License Application (BLA)	Marketing authorization application
Marketing authorization approvals—“Facilitated Pathways” *	**Promoting Clinical Development**	Breakthrough Therapy Designation	Priority medicines (PRIME) scheme
	**Reducing MA review timeline**	Fast Track Designation Priority Review	Accelerated assessment
	**MAs without comprehensive clinical studies**	NA	MA under exceptional circumstances
	**MAs prior clinical confirmatory studies**	Accelerated Approval	Conditional marketing authorization
	**MAs during recognized emergency public health threats**	Emergency use authorization	

^*^“Facilitated Pathway” term is used to collectively describe all the available expedited, accelerated, emergency use and conditional pathways

## Study Aim and Objectives

The aim of this study was to compare and contrast the EU and US regulations, processes, and outcomes pertaining to the granting accelerated MAs for Covid-19 vaccines and treatments with a view to determine how effective these regulations were in delivering safe medicines in a timely manner.

The study's objectives were as follows:

i. To identify and compare all official marketing authorizations granted by EMA and USFDA for Covid-19 vaccines and treatments, and
ii. To identify and compare the corresponding implemented regulations and their effectiveness.

## Methodology

### Identification and Comparison of MAs for Covid-19 Vaccines and Treatments in EU and USA

In order to address the first objective, the official EMA and USFDA websites [2–4] were searched to find all Covid-19 vaccines and treatments that were officially approved during the Covid-19 pandemic on an accelerated basis. In the EU, all Covid-19 MAs had been granted through the EMA centralized procedure. The inclusion and exclusion criteria listed in Table 4 were used to extract relevant data published by 28 February 2022, giving a 2-year timeframe since the first WHO declaration of a pandemic.

**Table 4:** Inclusion and exclusion criteria for Covid-19 vaccines and treatments MAs

Inclusion criteria	Exclusion criteria
• Initial USFDA and centralized EMA official authorizations for Covid-19 vaccines and treatments by either standard marketing authorization or any of the facilitated pathways like US emergency use authorization or EU conditional marketing authorization • Covid-19 vaccines and treatments including drugs and non-vaccine biological products which contain new entities i.e., new active chemical or biological substances • Covid-19 treatment new indication authorizations for products which contain previously authorized entities	• Investigational new drugs which are being tested in clinical trials for Covid-19 and/or submitted for marketing authorization application but have not yet received official marketing authorization from USFDA or EMA • Covid-19 vaccines and treatments that are under research and development and have received EMA advice or under evaluation/rolling review or review under Article 5(3) of the Regulation (EC) No 726/2004 by EMA but not yet received official authorization • Authorized medical devices related to Covid-19 such as in-vitro diagnostic test kits, protective equipment, ventilators, etc • Emergency use authorizations of products to be used during the Covid-19 pandemic but not indicated for Covid-19 treatment (e.g., general anesthetics or replacement solutions) • Any revoked Covid-19 vaccine or treatment authorizations during the study period

The European Public Assessment Reports (EPARs)^1^ for initial authorizations and US authorization letters with accompanying review memorandum^2^ documents were accessed for each identified Covid-19 vaccine and treatment MA “Covid-19 MA.”

The following data were extracted into excel from EPARs and US review memorandum documents: product category (vaccine or treatment), entity status (new entity or new indication of previously authorized entity), therapeutic group of entity (ATC code if available), approved indication(s), application submission date, initial authorization date, authorization pathway (standard or any facilitated pathway), number and design of accepted pivotal clinical trials for safety and efficacy evaluation, and issued post-authorization obligations.

For the Covid-19 MAs approved by facilitated pathways, the conversion status to standard authorization was also recorded. Additional searches were conducted in “Drugs@FDA: FDA-Approved Drug” and “Licensed Biological Products” databases [35, 36] to determine if any US Covid-19 MAs were based on standard NDAs or BLAs. If so, standard respective submission and approval dates were extracted from the corresponding “Summary Basis of Regulatory Action” or “Summary Review” documents.

Based on the extracted details, Covid-19 MAs were compared for the following points:

- Number and type of Covid-19 Medicines authorized by EMA and USFDA,
- Common Covid-19 medicines which were authorized by both EMA and USFDA,
- Approval timelines of Covid-19 MAs, calculated as the period between official application submission and initial authorization dates,
- Used authorization pathways (standard or facilitated pathways),
- Status of conversion to standard authorization,
- Variety of therapeutic groups,
- Approved indications,
- Accepted pivotal clinical trials and type of issued post-authorization clinical requirements.

### Identification and Comparison of the Utilized EU and US Regulations for Covid-19 MAs

In order to address objective 2, the following searches were undertaken:

1. European Union’s (Eudralex^3^—Volume 1—Pharmaceutical legislation for medicinal products for human use) official website [38], which publishes the rules and regulations governing medicinal products in the EU. (The volume containing guidelines is not considered since guidelines are not legally binding documents).
2. Title 21 of US Code of Federal Regulations [39] and Titles 21 and 42 of US Code [40], which govern food, drugs, and biologics in the United States.^4^

Using the inclusion and exclusion criteria listed in Table 5, the relevant regulations were accessed via official websites and then screened and reviewed to extract the regulations related to the following:

i. Procedures, approval timelines, clinical requirements, and post-authorization requirements for marketing authorization of new vaccines and treatments intended for viral diseases.
ii. Procedures and approval timelines for the approval of new therapeutic indications of already-approved entities.
iii. Criteria, procedures, and approval timelines for the used facilitated pathways or designations for Covid-19 MAs.

**Table 5:** Inclusion and exclusion criteria for EU and US utilized regulations

Inclusion criteria	Exclusion criteria
Legislation related to: • Marketing authorizations of medicines for human use • Marketing authorizations of new drugs and biologics • Variation approval for new indication addition of previously authorized entities • Facilitated pathways used in marketing authorizations of Covid-19 vaccines and treatments • Only procedures related to marketing authorization	Legislation related to: • Marketing authorizations of medicines for veterinary use • MAs of other product types not related to Covid-19 treatments and vaccines e.g., medical devices, herbal, orphan medicines, food, cosmetics, etc • Other lifecycle management activities of medicines like registration renewals • Facilitated pathways not used in marketing authorizations of Covid-19 vaccines and treatments • All other procedures such as conducting clinical trials, clinical trials applications/INDs, medicines manufacturing, distribution, advertising, pharmacovigilance, pricing, etc

The utilized regulations and processes used for approval of Covid-19 vaccines and treatments during the pandemic were compared between USFDA and EMA.

The effectiveness of the utilized regulations in delivering safe medicines in a timely manner during an emergency situation was assessed by observing them against the following time and safety standards:

1. If utilizing such regulations for Covid-19 MAs resulted in faster approval timelines compared to standard MAs.
2. If the utilized regulations were flexible enough to manage the process of granting emergency approvals while maintaining strict requirements.
3. If the clinical trials supporting the Covid-19 MAs were in line with the regulations and requirements.
4. If the regulatory authorities were able to make a comprehensive assessment of all data supporting the Covid-19 MAs despite fast approval timelines.

## Results

### Identification and Comparison of MAs for Covid-19 Vaccines and Treatments in EU and USA

#### Number and Type of MAs for Covid-19 Vaccines and Treatments

- By 28 February 2022, the EMA and USFDA had granted a total of 12 and 14 MAs for Covid-19 vaccines and treatments, respectively.
- The EMA authorized five vaccines and seven treatments, and the USFDA authorized three vaccines and eleven treatments.
- All vaccines were new entities. Two of the seven EMA Covid-19 treatments and two of the 11 USFDA Covid-19 treatments were previously authorized entities for which a new Covid-19 treatment indication was granted; the rest were new entities.
- Eight of all available Covid-19-related products were approved by both USFDA and EMA (three vaccines and five treatments), four products were approved only by EMA (two vaccines and two treatments), and six products were approved only by US (six treatments).

**Figure 1:**
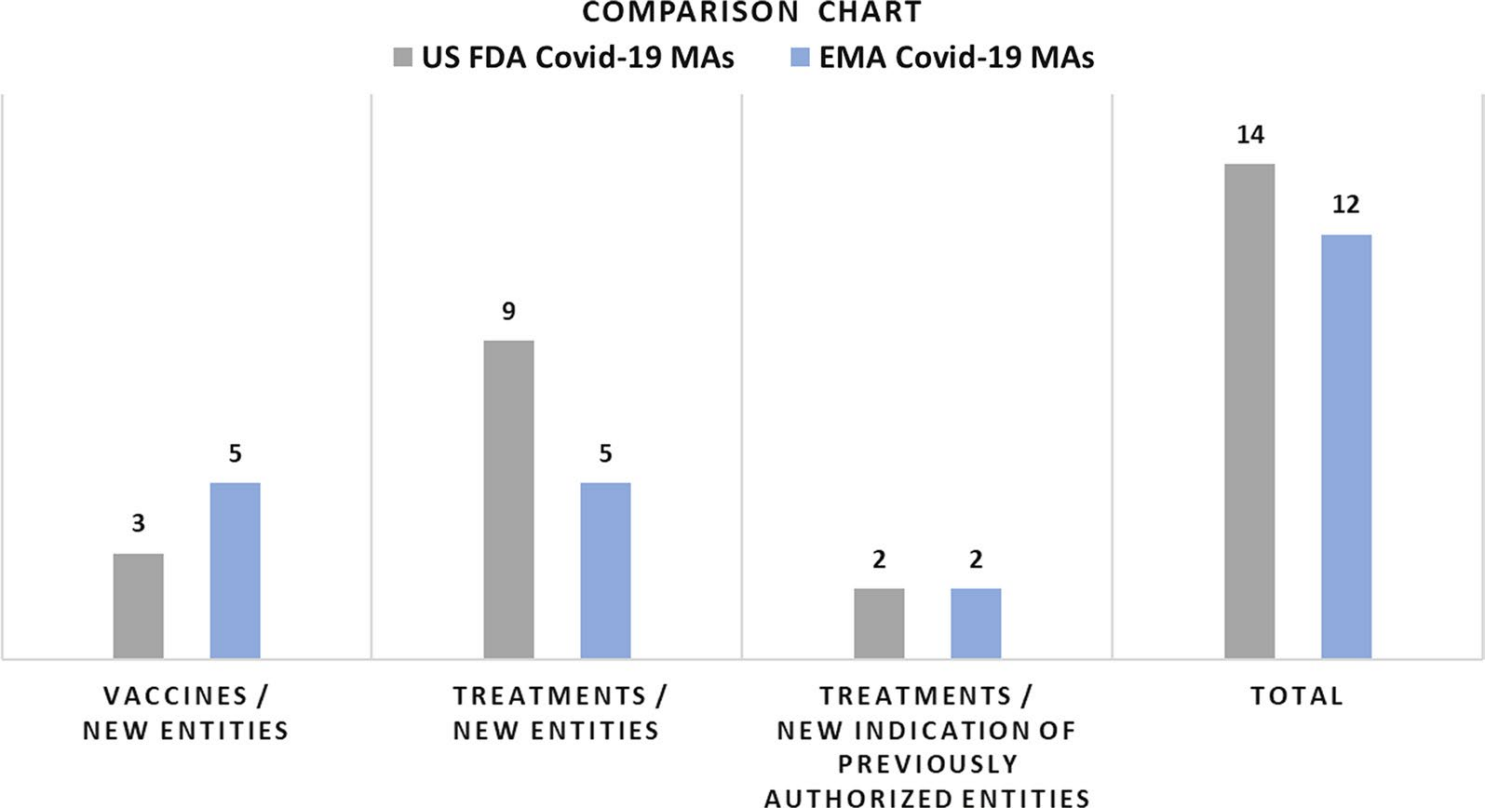
Comparison between EMA and USFDA in number and type of Covid-19 MAs.

Table 6 lists the EMA and USFDA authorized Covid-19 vaccines and treatments, alongside the accessed references of initial authorization EPARs and US emergency authorization letters with accompanying review memorandums. Figure 1 illustrates the comparison between EMA and USFDA in number and type of Covid-19 MAs.

**Table 6:** **Covid-19 vaccines and treatments authorized by EMA and USFDA by 28 Feb 2022**

Entity/product name	Product category	Entity status	Sponsor in USA / MAH in Europe	References
**Covid-19 Vaccines/Treatments authorized by both USFDA and EMA**				
Pfizer- BioNTech COVID-19 Vaccine/[Comirnaty]	Vaccine	New Entity	USA: Pfizer, on behalf of Pfizer and BioNTech) Europe: BioNTech Manufacturing GmbH	USFDA Authorization Letter/Review memo. [57, 58] EMA EPAR [59]
Janssen COVID-19 Vaccine	Vaccine	New Entity	USA: Janssen Biotech, Inc Europe: Janssen-Cilag International NV	USFDA Authorization Letter/Review memo. [60, 61] EMA EPAR [62]
Moderna COVID-19 Vaccine / [Spikevax]	Vaccine	New Entity	USA: Moderna TX Europe: Moderna Biotech Spain S.L	USFDA Authorization Letter/Review memo. [63, 64] EMA EPAR [65]
Casirivimab co-packaged with Imdevimab / [Ronapreve in EMA, REGEN-COV in USFDA]	Treatment	New Entity	USA: Regeneron Pharmaceuticals, Inc Europe: Roche Registration GmbH	USFDA Authorization Letter/Review memo. [66, 67] EMA EPAR [68]
Remdesivir / [Veklury]	Treatment	New Entity	USA: Gilead Sciences, Inc Europe: Gilead Sciences Ireland UC	USFDA Authorization Letter/Review memo. [69, 70] EMA EPAR [71]
Sotrovimab / [Xevudy]	Treatment	New Entity	USA: Pfizer, Inc.’s (Pfizer) Europe: Pfizer Europe MA EEIG	USFDA Authorization Letter/Review memo. [72, 73] EMA EPAR [74]
Nirmatrelvir co-packaged with ritonavir/[Paxlovid]	Treatment	New Entity	USA: GlaxoSmithKline Research & Development Limited Europe: GlaxoSmithKline	USFDA Authorization Letter/Review memo. [75, 76] EMA EPAR [77]
Tocilizumab / [RoActemra in EMA, Actemra is USFDA]	Treatment	New indication of previously authorized entity	USA: Genentech, Inc.’s (Genentech) Europe: Roche Registration GmbH	USFDA Authorization Letter/Review memo. [78, 79] EMA EPAR [80]
**Covid-19 Vaccines/Treatments authorized by EMA only**				
AstraZeneca COVID-19 Vaccine/[Vaxzevria]	Vaccine	New Entity	Europe: AstraZeneca AB	EMA EPAR [81]
COVID-19 Vaccine (recombinant, adjuvanted) / [Nuvaxovid]	Vaccine	New Entity	Europe: Novavax CZ, a.s	EMA EPAR [82]
Aanakinra / [Kineret]	Treatment	New indication of previously authorized entity	Europe: Swedish Orphan Biovitrum AB (publ)	EMA EPAR [83]
Regdanvimab / [Regkinora]	Treatment	New Entity	Europe: Celltrion Healthcare Hungary Kft	EMA EPAR [84]
**Covid-19 Vaccines/Treatments authorized by US FDA only**				
Molnupiravir	Treatment	New Entity	USA: Merck Sharp & Dohme Corp.’s (Merck)	USFDA Authorization Letter/Review memo. [85, 86]
Tixagevimab co-packaged with Cilgavimab / [Evusheld]	Treatment	New Entity	USA: AstraZeneca Pharmaceuticals LP’s (AstraZeneca)	USFDA Authorization Letter/Review memo. [87, 88]
Bamlanivimab + Etesevimab	Treatment	New Entity	USA: Eli Lilly and Company	USFDA Authorization Letter/Review memo. [89, 90]
Baricitinib / [Olumiant]	Treatment	New indication of previously authorized entity	USA: Eli Lilly and Company	USFDA Authorization Letter/Review memo. [91, 92]
COVID-19 convalescent plasma	Treatment	New Entity*	USA: The Office of the Assistant Secretary for Preparedness and Response (ASPR) U.S. Department of Health and Human Services (HHS)	USFDA Authorization Letter/Review memo. [93, 94]
Bebtelovimab	Treatment	New Entity	USA: Eli Lilly and Company	USFDA Authorization Letter/Review memo. [95, 96]

^*^The content is not new chemical or biological entity itself. However, the product was not available previously in any form and for any other condition

#### Approval Timelines, Facilitated Pathways, and Conversion Status to Standard Approval

- All USFDA initial approvals were under the Emergency Use Authorization pathway with some having additional Fast Track Designation.
- Seven EMA approvals were under the Conditional MA pathway, and five were under Standard MAs. All EMA new entity approvals had gone through a rolling review procedure following Covid-19 EMA pandemic Task Force (COVID-ETF) recommendation.
- None of EMA conditional approvals had been converted to standard approval by 28 February 2022; in USA, three of all emergency approvals had been converted to standard approval.

#### Approval Timeline Comparison (EMA Conditional MA vs. USFDA Emergency Use Authorization):

The median approval timeline was 24 days for all Covid-19 products authorized under the EMA’s Conditional MA pathway, whereas the median approval timeline for the USFDA’s Emergency Use Authorization pathway was 36 days.

#### Approval Timeline Comparison (For Covid-19 MAs authorized by both EMA and USFDA):

- ○ The USFDA approved all new novel entities faster (median 23 days) than the EMA (median 28 days).
- ○ The USFDA approved new entity vaccines faster (median 21 days) than the EMA (median 24 days), yet the EMA approved new entity treatments faster (median 29 days) than the USFDA (median 40 days).
- ○ A marked difference was also found between the approval timelines for new indication addition to already-authorized entities, with USFDA authorization faster (median 65 days) than EMA (median 133 days).
- ○ The USFDA approved all Covid-19 vaccines and treatments before the EU. Sponsors submitted applications first to USFDA before submitting to the EMA for seven out of eight cases.

**Figure 2:**
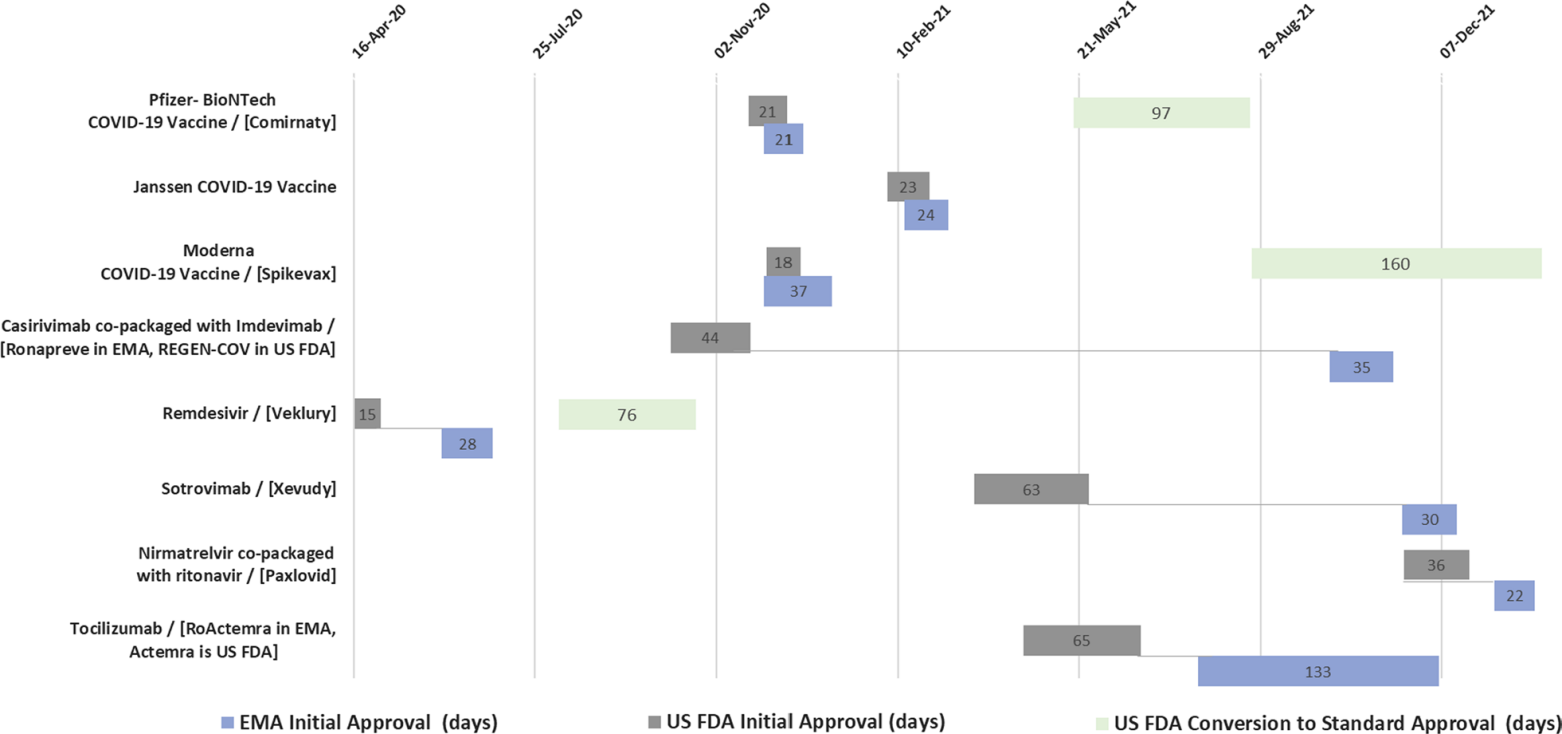
Comparison between EMA and USFDA in approval timelines for Covid-19 MAs authorized by both authorities.

Figure 2 and Tables 7 and 8 illustrate the details of approval timelines and pathways.

**Table 7:** Comparison between EMA and USFDA in Covid-19 MAs Approval Timelines and Pathways

		European Medicines Agency					US Food and Drug Administration				
Entity/Common name(s)	Entity status/product type	Approval pathway(s)	Submission date	Approval date	Approval timeline (days)	Conversion to standard approval	Approval pathway(s)	Submission date	Approval date	Approval timeline (days)	Conversion to standard approval
**Authorized both by EMA and USFDA**											
**Pfizer- BioNTech COVID-19 Vaccine**/[Comirnaty]	New Entity Vaccine	Conditional MA Rolling Review	30-Nov-20	21-Dec-20	21	**No**	Emergency Use Authorization Fast Track Designation	20-Nov-20	11-Dec-20	21	**Converted to Standard Approval**
**Janssen COVID-19 Vaccine**	New Entity Vaccine	Conditional MA Rolling Review	15-Feb-21	11-Mar-21	24	**No**	Emergency Use Authorization	04-Feb-21	27-Feb-21	23	**No**
**Moderna COVID-19 Vaccine** / [Spikevax]	New Entity Vaccine	Conditional MA Rolling Review	30-Nov-20	06-Jan-21	37	**No**	Emergency Use Authorization Fast Track Designation	30-Nov-20	18-Dec-20	18	**Converted to Standard Approval**
**Casirivimab co-packaged with Imdevimab** / [Ronapreve in EMA, REGEN-COV in USFDA]	New Entity Treatment	Standard MA Rolling Review	08-Oct-21	12-Nov-21	35	**Already approved by standard pathway**	Emergency Use Authorization	08-Oct-20	21-Nov-20	44	**No**
**Remdesivir** / [Veklury]	New Entity Treatment	Conditional MA Rolling Review	05-Jun-20	03-Jul-20	28	**No**	Emergency Use Authorization Fast Track Designation	16-Apr-20	01-May-20	15	**Converted to Standard Approval**
**Sotrovimab** / [Xevudy]	New Entity Treatment	Standard MA Rolling Review	17-Nov-21	17-Dec-21	30	**Already approved by standard pathway**	Emergency Use Authorization	24-Mar-21	26-May-21	63	**No**
**Nirmatrelvir co-packaged with ritonavir**/[Paxlovid]	New Entity Treatment	Conditional MA Rolling Review	7-Jan-22	28-Jan-22	21	**No**	Emergency Use Authorization Fast Track Designation	16-Nov-21	22-Dec-21	36	**No**
**Tocilizumab** / [RoActemra in EMA, Actemra is USFDA]	New Indication Treatment	Standard Variation Approval	27-Jul-21	07-Dec-21	133	**Already approved by standard pathway**	Emergency Use Authorization	20-Apr-21	24-Jun-21	65	**No**
**Authorized only by EMA**											
**AstraZeneca COVID-19 Vaccine**/[Vaxzevria]	New Entity Vaccine	Conditional MA Rolling Review	11-Jan-21	29-Jan-21	18	**No**	**NA**				
**COVID-19 Vaccine (recombinant, adjuvanted) **/ [Nuvaxovid]	New Entity Vaccine	Conditional MA Rolling Review	16-Nov-21	20-Dec-21	34	**No**					
**Aanakinra** / [Kineret]	New Indication Treatment	Standard Variation Approval	08-Jul-21	17-Dec-21	162	**Already approved by standard pathway**					
**Regdanvimab** / [Regkinora]	New Entity Treatment	Standard MA Rolling Review	01-Oct-21	12-Nov-21	42	**Already approved by standard pathway**					
**Authorized only by USFDA**											
**Molnupiravir**	New Entity Treatment	**NA**					Emergency Use Authorization	08-Oct-21	23-Dec-21	76	**No**
**Tixagevimab co-packaged with Cilgavimab** / [Evusheld]	New Entity Treatment						Emergency Use Authorization	30-Sep-21	08-Dec-21	69	**No**
**Bamlanivimab + Etesevimab**	New Entity Treatment						Emergency Use Authorization	16-Nov-20	09-Feb-21	85	**No**
**Baricitinib** / [Olumiant]	New Indication Treatment						Emergency Use Authorization	15-Oct-20	19-Nov-20	35	**No**
**COVID-19 convalescent plasma**	New Entity Treatment						Emergency Use Authorization	NA	23-Aug-20	NA	**No**
**Bebtelovimab**	New Entity Treatment						Emergency Use Authorization	07-Jan-22	11-Feb-22	35	**No**

**Table 8:** Continued USFDA Approval Timelines and Pathways for Covid-19 MAs Converted to Standard Approval

Entity/Common Name(s)	Entity status/product type	Application type	Additional facilitated pathway(s)	Standard application submission date	Standard approval date	Approval timeline (days)	Reference for NDA/BLA summary basis of regulatory action”/“summary review”
**Pfizer- BioNTech COVID-19 Vaccine**/[Comirnaty]	New Entity Vaccine	Biological License Application (BLA)	Priority Review for Clinical review	18-May-21	23-Aug-21	97	[97]
**Moderna COVID-19 Vaccine**/ [Spikevax]	New Entity Vaccine	Biological License Application (BLA)	Priority Review	24-Aug-21	31-Jan-22	160	[98]
**Remdesivir **/ [Veklury]	New Entity Treatment	New Drug Application (NDA)	Priority Review	07-Aug-20	22-Oct-20	76	[99]

#### Therapeutic Groups, Approved Indications, and Accepted Pivotal Clinical Trials

**Figure 3:**
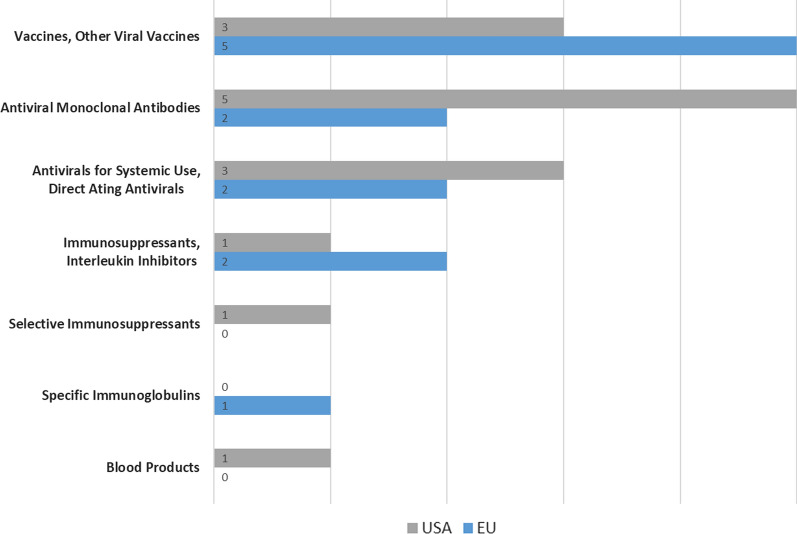
Comparison between therapeutic groups of USFDA and EMA Covid-19 MAs.

- The Covid-19 vaccines and treatments authorized in EU and USA varied between different pharmacotherapeutic groups as shown in Fig. 3.
- EMA and USFDA authorized vaccines had the same approved indications, but indications of most of treatments differed. For example, Casirivimab co-packaged with Imdevimab was authorized for treatment and prevention in the EU but only for treatment in the USA. Other examples include Nirmatrelvir co-packaged with ritonavir, and Tocilizumab, which the EMA authorized only for use in adults, but the USFDA authorized them for use in adults and children. Table 9 lists the statements of all approved indications.
- All clinical trials supporting US and EU applications for MA of Covid-19 indications were randomized and placebo controlled.
- Two or more pivotal phase 3 clinical trials underpinned only three of twelve EMA authorizations and two of fourteen USFDA authorizations for Covid-19 indications *(a minimum of two is recommended *[42]*)*. The remaining approvals were based on only one single pivotal phase 3 clinical trial. Two USFDA authorizations were approved without a pivotal phase 3 clinical trial, with one of them authorized based on totality of scientific evidence and small multiple trials, and the other on one phase 1/2 trial.
- Only six of 12 clinical trials supporting EMA applications and four of 14 clinical trials supporting USFDA applications for Covid-19 indications had been completed, with the remainder still “ongoing” at the time of authorization.
- The EMA issued post-authorization clinical obligations for Covid-19 indications which received conditional authorization, with most being obligations to continue the ongoing clinical trials. The USFDA granted emergency use authorizations based on the sponsor’s plans to continue the ongoing trials for some applications and by identifying outstanding issues/data gaps to provide additional confirmatory studies or requirements for some other applications.

**Table 9:** Therapeutic groups, approved indications, accepted pivotal clinical trials, and post-authorization obligations for EMA and USFDA Covid-19 MAs

Region	Entity/Product name	Pharmaco-therapeutic group (WHO ATC Code)	Approved initial indications	Pivotal Phase 3 Clinical studies supporting the MA application				Post-authorization clinical obligations
				Number of trials + (Study Code)	Characteristics	Planned participants number	Status	
**Authorized both by EMA and USFDA**								
**EU**	**Pfizer- BioNTech COVID-19 Vaccine/[Comirnaty]**	J07BX03 “Vaccines, other viral vaccines”	Active immunization to prevent COVID-19 caused by SARS-CoV-2 virus, in individuals 16 years of age and older	1 (C4591001)	Randomized, multinational, placebo-controlled, observer-blind	44,000	Ongoing	Completion of ongoing trials *(as obligation for conditional authorization)*
**USA**								Completion of ongoing trials *(EUA based on sponsor’s plans for continuation) (c)*
**EU**	**Janssen COVID-19 Vaccine**	J07BX03 “Vaccines, other viral vaccines	Active immunization to prevent COVID-19 caused by SARS-CoV-2 in individuals 18 years of age and older	1^(a)^ (VAC31518COV3001)	Randomized, placebo-controlled, double-blind	40,000	Ongoing	Completion of ongoing trials *(as obligation for conditional authorization)*
**USA**								Completion of ongoing trials *(EUA based on sponsor’s plans for continuation) *^*(c)*^
**EU**	**Moderna COVID-19 Vaccine /** **[Spikevax]**	J07BX03 “Vaccines, other viral vaccines	Active immunization to prevent COVID-19 caused by SARS-CoV-2 virus in individuals 18 years of age and older	1 (mRNA-1273-P301	Randomized, stratified, placebo- controlled, observer-blind	30,000	Ongoing	Completion of ongoing trials *(as obligation for conditional authorization)*
**USA**								Completion of ongoing trials *(EUA based on sponsor’s plans for continuation) *^*(c)*^
**EU**	**Casirivimab co-packaged with Imdevimab /** **[Ronapreve in EMA, REGEN-COV in USFDA]**	J06BD “Antiviral monoclonal antibodies”	Treatment of COVID-19 in adults and adolescents aged 12 years and older weighing at least 40 kg who do not require supplemental oxygen and who are at increased risk of progressing to severe COVID-19 + Prevention of COVID-19 in adult patients and in adolescent patients aged 12 years and older weighing at least 40 kg	1 (treatment indication) (COV-2067) + 1 (prevention indication) (COV-2069)	Randomized, Placebo-controlled, Double-Blind	4567 + 3029	Completed	Not available (Standard Authorization)
**USA**			Treatment of mild-to-moderate coronavirus disease 2019 (COVID-19) in adults and pediatric patients (12 years of age and older weighing at least 40 kg) with positive results of direct SARS-CoV-2 viral testing, and who are at high risk for progressing to severe COVID-19 and/or hospitalization	0 (Based on phase 1/2 trial) (COV-2067)		799	Ongoing	Not available
**EU**	**Remdesivir /** **[Veklury]**	J05AB16 “Antivirals for systemic use, direct acting antivirals”	Treatment of coronavirus disease 2019 (COVID-19) in adults and adolescents (aged 12 years and older with body weight at least 40 kg) with pneumonia requiring supplemental oxygen	1^(a)^ (CO-US-540–5776/Protocol No. 20–0006)	Randomized, placebo-controlled, double-blind	1063	Ongoing	Completion of ongoing trials *(as obligation for conditional authorization*
**USA**			Treatment of hospitalized patients with severe 2019 coronavirus disease (COVID-19)	2^(a)^ (Protocol No.20–0006 /NCT04280705 + Wang et al. (2020) Trial/NCT0425765)		1063 + 237	Ongoing + Terminated	Not available^(c)^
**EU**	**Sotrovimab /** **[Xevudy]**	J06BD “Antiviral monoclonal antibodies”	Treatment of adults and adolescents (aged 12 years and over and weighing at least 40 kg) with coronavirus disease 2019 (COVID-19) who do not require oxygen supplementation and who are at increased risk of progressing to severe COVID-19	1^(a)^ (COMET-ICE)	Randomized, placebo-controlled, double-blinded	1057	Completed	Not available (Standard Authorization)
**USA**			Treatment of mild-to-moderate coronavirus disease 2019 (COVID-19) in adults and pediatric patients (12 years of age and older weighing at least 40 kg) with positive results of direct SARS-CoV-2 viral testing, and who are at high risk for progression to severe COVID-19, including hospitalization or death				Ongoing	Completion of ongoing Trials *(As identified outstanding issues/data gaps)*
**EU**	**Nirmatrelvir co-packaged with ritonavir/[Paxlovid]**	“Antiviral for systemic use”	Treatment of coronavirus disease 2019 (COVID-19) in adults who do not require supplemental oxygen and who are at increased risk for progressing to severe COVID-19	1 (EPIC-HR, C4671005)	Randomized, placebo-controlled, double-blinded	3100	Completed	Additional confirmatory requirements/studies *(As legally binding measures and recommendations)*
**USA**			Treatment of mild-to-moderate COVID-19 in adults and pediatric patients (12 years of age and older weighing at least 40 kg) with positive results of direct SARS-CoV-2 viral testing, and who are at high risk2for progression to severe COVID-19, including hospitalization or death			2246	Ongoing	Additional confirmatory requirements/studies in addition to completion of ongoing trial *(As identified outstanding issues/data gaps)*
**EU**	**Tocilizumab /** **[RoActemra in EMA, Actemra is USFDA]**	L04AC07 “Immunosuppressants, Interleukin inhibitors”	Treatment of coronavirus disease 2019 in adults who are receiving systemic corticosteroids and require supplemental oxygen or mechanical ventilation	4 (COVACTA + EMPACTA + REMDACTA + RECOVERY)	3 Randomized, placebo-controlled, double-blinded 1 randomized, controlled, open-label, platform trial (RECOVERY)	452 + 388 + 649 + 4116	Completed	Not available (Standard Authorization)
**USA**			Treatment of coronavirus disease 2019 (COVID-19) in hospitalized adults and pediatric patients (2 years of age and older) who are receiving systemic corticosteroids and require supplemental oxygen, non-invasive or invasive mechanical ventilation, or extracorporeal membrane oxygenation (ECMO)					Not available
**Authorized only by EMA**								
**EU**	**AstraZeneca COVID-19 Vaccine/[Vaxzevria]**	J07BX03 “Vaccines, other viral vaccines	Active immunization to prevent COVID-19 caused by SARS-CoV-2, in individuals 18 years of age and older	2 (COV002 + COV003)	Both randomized, controlled, participant blind	12,390 + 10,000	Both Ongoing	Completion of ongoing trials *(as obligation for conditional authorization)*
**EU**	**COVID-19 Vaccine (recombinant, adjuvanted) /** **[Nuvaxovid]**	J07BX03 “Vaccines, other viral vaccines	Active immunization to prevent COVID-19 caused by SARS-CoV-2 in individuals 18 years of age and older	2 (2019nCoV-302 + 2019nCoV-301)	Both randomized, controlled, observer blind	15,000 + 30,000	Both Ongoing	Not available *(there is additional recommendation only for clinical efficacy)*^*(b)*^
**EU**	**Aanakinra /** **[Kineret]**	L04AC03 “Immunosuppressants, Interleukin inhibitors”	Treatment of coronavirus disease 2019 (COVID-19) in adult patients with pneumonia requiring supplemental oxygen (low- or high-flow oxygen) who are at risk of progressing to severe respiratory failure determined by plasma concentration of soluble urokinase plasminogen activator receptor (suPAR) ≥ 6 ng/ml	1 (SAVE-MORE)	Randomized, placebo-controlled, double-blind	1060	Completed	Not available (Standard Authorization)
**EU**	**Regdanvima /** **[Regkinora]**	J06BB “Specific immunoglobulins”	Treatment of adults with coronavirus disease 2019 (COVID-19) who do not require supplemental oxygen and who are at increased risk of progressing to severe COVID-19	1 (CT-P59 3.2)	Randomized, placebo-controlled, double-blind	Part 1: 327 Part 2: 1315	Completed	Not available (Standard Authorization)
**Authorized only by USFDA**								
**USA**	**Molnupiravir**	“Antiviral for systemic use”	Treatment of mild-to-moderate COVID-19 in adults who are at high risk for progression to severe COVID-19, including hospitalization or death and for whom alternative COVID-19 treatment options authorized by FDA are not accessible or clinically appropriate	1^(a)^ (MK-4482–002)	Randomized, placebo-controlled, double-blinded	1433	Completed	Additional confirmatory requirements/studies *(As identified outstanding issues/data gaps)*
**USA**	**Tixagevimab co-packaged with Cilgavimab /** **[Evusheld]**	J06BD03 “Antiviral monoclonal antibodies”	Pre-exposure prophylaxis of COVID-19 in adults and pediatric individuals (12 years of age and older weighing at least 40 kg): • Who are not currently infected with SARS-CoV-2 and who have not had a known recent exposure to an individual infected with SARS-CoV-2 **and** • Who have moderate to severe immune compromise due to a medical condition or receipt of immunosuppressive medications or treatments **and** may not mount an adequate immune response to COVID-19 vaccination **or** • For whom vaccination with any available COVID-19 vaccine, according to the approved or authorized schedule, is not recommended due to a history of severe adverse reaction (e.g., severe allergic reaction) to a COVID-19 vaccine(s) and/or COVID-19 vaccine component(s)	1^(a)^ (PROVENT)	Randomized, placebo-controlled, double-blinded	5197	Ongoing	Additional confirmatory requirements/studies in addition to completion of ongoing trial *(As identified outstanding issues/data gaps)*
**USA**	**Bamlanivimab + Etesevimab**	“Antiviral monoclonal antibodies”	Treatment of mild-to-moderate coronavirus disease 2019 (COVID-19) in adults and pediatric patients (12 years of age and older weighing at least 40 kg) with positive results of direct SARS-CoV-2 viral testing, and who are at high risk for progressing to severe COVID-19 and/or hospitalization	1^(a)^ (J2W-MC-PYAB/BLAZE-1)	Randomized, placebo-controlled, double-blinded	3890	Ongoing	Not available
**USA**	**Baricitinib /** **[Olumiant]**	L04AA37 “Selective immunosuppressants”	Treatment of COVID-19 in hospitalized adults and pediatric patients 2 years of age or older requiring supplemental oxygen, non-invasive or invasive mechanical ventilation, or ECMO (in combination with remdesivir)	1^(a)^ (Protocol No 20–0006 (ACTT-2) NCT04401579)	Randomized, placebo-controlled, double-blinded	1033	Completed	Not available
**USA**	**COVID-19 convalescent plasma**	“Other blood products”	Treatment of hospitalized patients with COVID-19	Multiple small trials and criteria ^(d)^	NA	NA	Completed	Additional confirmatory requirements/studies *(As recommendation)*
**USA**	**Bebtelovimab**	Antiviral monoclonal antibodies	Treatment of mild-to-moderate coronavirus disease 2019 (COVID-19) in adults and pediatric patients (12 years of age and older weighing at least 40 kg) with positive results of direct SARS-CoV-2 viral testing, and who are at high risk for progression to severe COVID-19, including hospitalization or death, and for whom alternative COVID-19 treatment options approved or authorized by FDA are not accessible or clinically appropriate	0 (Based on phase 1/2 trial) PYAH; BLAZE-4 NCT04634409	Randomized, placebo-controlled, double-blind, single dose trial	1416	Ongoing	Additional confirmatory requirements/studies in addition to completion of ongoing trial *(As identified outstanding issues/data gaps*

^a^There are additional phase 3/supportive clinical trials. However, they are not considered pivotal in the application to support the main approved indication and/or results are not provided.

^b^Main obligations to complete post-authorization measures for conditional marketing authorization are related to quality of the product and not related to clinical trials.

^c^US FDA requested new post-marketing confirmatory studies. However, these requirements were issued after receiving BLA standard approval and not with initial emergency use authorization.

^d^Authorized based on (1) historical evidence using convalescent plasma in prior outbreaks of respiratory viruses, (2) certain pre-clinical evidence; (3) results from small clinical trials and observational studies of convalescent plasma conducted during the current outbreak; and (4) data obtained from the ongoing National Expanded Access Treatment Protocol (EAP) sponsored by the Mayo Clinic.

**Figure 4:**
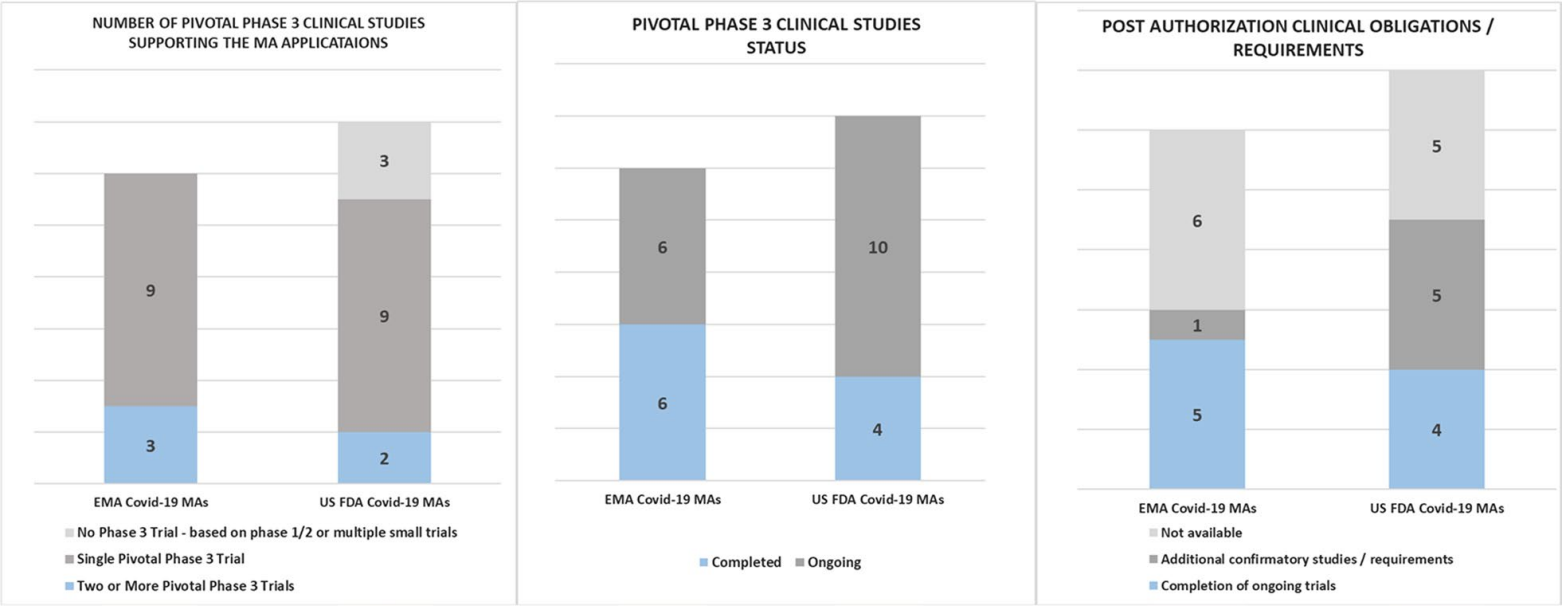
Comparison between EMA and USFDA in the accepted number/status of pivotal clinical trials and post-authorization clinical obligations.

Figure 4 illustrates these findings and Table 9 lists all the therapeutic groups, approved indications, accepted pivotal clinical trials, and post-authorization obligations for EU and US Covid-19 MAs.

### Identification and Comparison of the Utilized EU and US Regulations for Covid-19 MAs

Following the processes outlined in Sect. "Identification and Comparison of the Utilized EU and US Regulations for Covid-19 MAs", the identified EU and US regulations utilized in MAs of Covid-19 vaccines and treatments are listed in Tables 10 and 11, respectively. Key points of these regulations are summarized in Tables 12 and 13, respectively.

**Table 10:** Identified EU legislations utilized in marketing authorization of Covid-19 vaccines and treatments

Identified EU legislations
• Directive 2001/83/EC of the European Parliament and of the Council of 6 November 2001 on the Community code relating to medicinal products for human use [30]
• Regulation (EC) No 726/2004 of the European Parliament and of the Council of 31 March 2004 laying down Community procedures for the authorization and supervision of medicinal products for human and veterinary use and establishing a European Medicines Agency [31]
• Regulation (EC) No 1901/2006 of the European Parliament and of the Council of 12 December 2006 on medicinal products for pediatric use and amending Regulation (EEC) No 1768/92, Directive 2001/20/EC, Directive 2001/83/EC and Regulation (EC) No 726/2004 [100]
• Regulation (EC) No 1394/2007 of the European Parliament and of the Council of 13 November 2007 on advanced therapy medicinal products [101]
• Commission Regulation (EC) No 1234/2008 of 24 November 2008 concerning the examination of variations to the terms of marketing authorizations for medicinal products for human use and veterinary medicinal products [102]
• Commission Regulation (EC) No 507/2006 of 29 March 2006 on the conditional marketing authorization for medicinal products for human use falling within the scope of Regulation (EC) No 726/2004 of the European Parliament and of the Council [32]
• Commission Regulation (EC) No 1662/95 of 7 July 1995 laying down certain detailed arrangements for implementing the Community decision-making procedures in respect of marketing authorizations for products for human or veterinary use [103]

**Table 11:** Identified US Regulations Utilized in Marketing Authorization of Covid-19 Vaccines and Treatments

Identified US regulations
• **21 CFR Part 314** Title 21 Food and Drugs > Chapter I Food and Drug Administration, Department of Health and Human Services > Subchapter D Drugs for Human Use > Part 314 Applications for FDA Approval to Market a New Drug [104]
• **21 CFR Part 601** Title 21 Food and Drugs > Chapter I Food and Drug Administration, Department of Health and Human Services > Subchapter F Biologics > Part 601 Licensing [23]
• **21 USC 355** Title 21 Food and Drugs > Chapter 9 Federal Food, Drug, And Cosmetic Act > Subchapter V Drugs and Devices > Part A Drugs and Devices > Sect. 355 New Drugs [105]
• **42 USC 262** Title 42 The Public Health and Welfare > Chapter 6A Public Health Service > Subchapter II General Powers And Duties > Part F Licensing Of Biological Products And Clinical Laboratories > Subpart 1 Biological product > Sect. 262 Regulation of Biological Products [106]
• **21 USC 356** Title 21 Food and Drugs > Chapter 9 Federal Food, Drug, And Cosmetic Act > Subchapter V Drugs and Devices > Part A Drugs and Devices > Sect. 356 Expedited approval of drugs for serious or life-threatening diseases or conditions [107]
• **21 USC 356b** Title 21 Food and Drugs > Chapter 9 Federal Food, Drug, And Cosmetic Act > Subchapter V Drugs and Devices > Part A Drugs and Devices > Sect. 356b Reports of post-marketing studies [108]
• **21 USC 360bbb-3** Title 21 Food and Drugs > Chapter 9 Federal Food, Drug, And Cosmetic Act > Subchapter V Drugs and Devices > Part E General Provisions Relating to Drugs and Devices > Sect. 360bbb-3 Authorization for medical products for use in emergencies [109]
• **21 USC 360bbb-3c** Title 21 Food and Drugs > Chapter 9 Federal Food, Drug, And Cosmetic Act > Subchapter V Drugs and Devices > Part E General Provisions Relating to Drugs and Devices > Sect. 360bbb-3c Expedited development and review of medical products for emergency uses [110]
• **21 USC 360bbb-4a** Title 21 Food and Drugs > Chapter 9 Federal Food, Drug, And Cosmetic Act > Subchapter V Drugs and Devices > Part E General Provisions Relating to Drugs and Devices > Sect. 360bbb-4a Priority review to encourage treatments for agents that present national security threats [111]
• **21 USC 379g to 379h-2** Title 21 Food and Drugs > Chapter 9 Federal Food, Drug, And Cosmetic Act > Subchapter VII General Authority > Part C Fees > Subpart 2 Fees relating to drugs > Sects. 379g to 379h-2 [112]

**Table 12:** Key Points from Identified EU Legislations

Search Area		Finding	Reference regulation
New Entities MAs	**Procedure(s)**	There are four types of procedures used in EU for marketing authorization of medicinal products: • Centralized Procedure • Decentralized Procedure • Mutual Recognition Procedure • National Procedure Procedure for Medicines intended for Viral Diseases: Centralized procedure is the compulsory route for the medicinal products which contain active substances targeted for viral diseases. Hence, only centralized procedure is applicable for Covid-19 MAs, in which one MA application is submitted to the EMA. Upon approval, final authorization is issued by the European Commission, which will be valid for all EU member states	**Directive 2001/83/EC** **Regulation (EC) No 726/2004 (for Centralized Procedure)** **Annex 1—Regulation (EC) No 726/2004**
	**Approval Timelines**	In standard cases, the EMA shall ensure the opinion of the Committee for Medicinal Products for Human Use is given within 210 days after receipt of a valid application in standard cases There are additional timelines for European Commission final decision-making procedure which take up to 52 days in case of approval decision *Note: In accelerated assessment procedure (not used for authorized Covid-19 vaccines and treatments), EMA shall ensure the opinion of the Committee for Medicinal Products for Human Use is given within 150 days instead of 210 days*	**Article 6 of Regulation (EC) No 726/2004** **Article 10 of Regulation (EC) No 726/2004,** **Commission Regulation (EC) No 1662/95** **Article 14(9) of Regulation (EC) No 726/2004**
	**Clinical Trials Requirements**	Each application for medicinal product authorization shall specifically and completely include the particulars and documents referred in Directive 2001/83/EC Clinical trials shall be done as “controlled clinical trials,” randomized and as appropriate versus placebo and versus an established medicinal product of proven therapeutic value. Any other design shall be justified *Note: Reference regulation section specifying the number of required pivotal phase 3 clinical trials is not found*	**Article 6 of Regulation (EC) No 726/2004** ** + ** **Directive 2001/83/EC**
	**Post-Authorization Studies**	After granting marketing authorization, where necessary the EMA may impose obligations to the MAH to conduct post-authorization clinical safety and/or efficacy studies	**Article 10(a) of Regulation (EC) No 726/2004**
	**Other Notes**	For therapeutic indications intended to population under 18 years old, the provisions of the Regulation (EC) No 1901/2006 on medicinal products for pediatric use are applied For advanced therapy medicinal products., the provisions of the Regulation (EC) No 1394/2007 are applied	**Regulation (EC) No 1901/2006** **Regulation (EC) No 1394/2007**
New Indication MAs of already-Authorized Entities	**Procedure**	New therapeutic indication addition is classified as major variation to the existing marketing authorization and requires type II variation application with all the required evidence and prior approval from the EMA and final decision from the European Commission ***Note:**** Only where there is dully recognized pandemic situation with respect to human influenza or human coronavirus, the Commission may, where certain pharmaceutical, non-clinical or clinical data are missing, exceptionally and temporarily accept a variation to the terms of a marketing authorization and the MAH shall submit the missing data within a time limit set by the relevant authority*	**Commission Regulation (EC) No 1234/2008** **Article (21) of Commission Regulation (EC) No 1234/2008**
	**Approval Timelines**	The EMA shall issue an opinion on the valid application within 90 days following its receipt in case of variations concerning a change to or addition of therapeutic indications. However, the agency may request supplementary information within a time limit set. In this case, the procedure shall be suspended until the supplementary information is provided Where the outcome of the assessment is favorable, the EMA shall transmit to the Commission its opinion within 15 days. The Commission, having regard to the opinion from the Agency and within 2 months (for new indication variation), shall amend where necessary the decision granting the marketing authorization The commission decision will be valid across all the EU member states	**Articles 16, 17 and 23(a) of Commission Regulation (EC) No 1234/2008**
Facilitated Pathways or Tools used for Covid-19 MAs	**Conditional MA**	**Criteria & Conditions**: Conditional MA is applicable to medicinal products: which aim at the treatment, the prevention or the medical diagnosis of seriously debilitating diseases or life-threatening diseases to be used in emergency situations, in response to public health threats duly recognized either by the World Health Organization or by the Community in the framework of Decision No 2119/98/EC designated as orphan medicinal products in accordance with Article 3 of Regulation (EC) No 141/2000 Conditional MA can be granted based on less complete data than is normally the case and subject to specific obligations to complete the ongoing studies or conduct new studies. However, the following requirements should be met: Risk benefit balance, as defined in Article 1(28a) of Directive 2001/83/EC should be positive It is likely that the applicant will be in a position to provide the comprehensive clinical data Unmet medical needs will be fulfilled The benefit to public health of the immediate availability on the market of the medicinal product concerned outweighs the risk inherent in the fact that additional data are still required There is special note saying that where conditional MAs are granted, they should be restricted to situations where only the clinical part of the application dossier is less complete than normal. Incomplete pre-clinical or pharmaceutical data should be accepted only in the case of a product to be used in emergency situations, in response to public health threats **Procedure:** The procedure for evaluating a conditional marketing authorization is the normal procedure laid down in Regulation (EC) No 726/2004 **Approval Timelines:** Conditional MA regulation does not specify any approval timelines **Validity:** Conditional MA will be valid for one year on a renewable basis	**Commission Regulation (EC) No 507/2006**
	**Rolling Review**	With Rolling Review process, the MAH can submit the data in many rolling review cycles as they become available from the ongoing studies to be reviewed by EMA before the formal application submission. Once the EMA’s human medicines committee decides that sufficient data are available, the MAH can submit the formal application This tool is introduced by EMA [49]. However, reference regulation section is not found	**Not available**

**Table 13:** Key Points from Identified US Regulations

Search Area		Finding	Reference regulation
New Entities MAs	**Procedure(s)**	For new drugs, New Drug Application (NDA) should be submitted and approved by the US FDA before entering the market For new biological products including vaccines, Biological License Application (BLA) should be submitted and approved by the US FDA before entering the market Standard pathway is followed requiring full set of efficacy and safety reports for new molecular entities and original BLAs. This is referred also as Sect. 505(b)(1) standard application pathway as per Federal Food, Drug, And Cosmetic Act	**21 CFR Part 314 + ** **21 USC 355** **21 CFR Part 601 + ** **42 USC 262** **21 USC 355 (b)(1) /** **FFD&C Sec 505(b)(1) **[21]
	**Approval Timelines**	In standard cases, FDA will review the New Drug Application within 180 days of the receipt “initial review cycle” and send back to the applicant either approval letter or complete response letter including the deficiencies. This timeline can be adjusted by mutual agreement between FDA and applicant In case of deficiencies and resubmission, there is additional review cycle of 2 months for class 1 minor resubmissions or 6 months for class 2 major resubmissions The regulations for licensing biological products do not specify BLA review timelines *Note: The Prescription Drug User Fee Act (PDUFA), which was first created by Congress in 1992 to authorize the FDA to collect fees from companies to expedite the drug approval process and reauthorized every 5 years, has performance goals for the review timelines to be 10 months from filing dates of both standard NDAs and BLA *[113]	**21 CFR Part 314.100 + ** **21 USC 355c** **21 CFR Part 314.110** **21 CFR Part 601 + ** **42 USC 262** *21 USC 379g to 379h-2*
	**Clinical Trials Requirements**	Applications for new molecular entities and original BLAs require full set of clinical efficacy and safety reports as per the standard pathway The design of the main clinical trials supporting the application should be controlled and adequate measures to be taken to minimize bias and assure comparability such as randomization and blinding Uncontrolled or partially controlled studies can be supportive. However, they cannot be accepted as sole basis for approval of effectiveness claims The number of required substantial or pivotal clinical trials is not specified by regulation	**21 USC 355 (b)(1)** **21 CFR 314.126**
	**Post-Authorization Studies**	The secretary shall request post-marketing studies upon agreement with the sponsor, which should be submitted within 1 year after the approval of the drug and annually tracked thereafter until the study is completed or terminated The post-marketing studies, or clinical trials can be requested at the time of approval or after approval if the Secretary becomes aware of new safety information	**21 USC 356b** **21 USC 355(o)**
New Indication MAs of already-Authorized Entities	**Procedure**	For post changes to an approved NDA or BLA, there are three types of changes: - Major changes which require supplement submission and prior approval before distribution. For these changes, expedited review can be requested for public health reasons - Moderate changes which require supplement submission at least 30 days before distribution - Minor Changes which can be described in annual report In standard cases, a new therapeutic indication addition to already-authorized drug or biological is considered a major change and needs supplemental application submission with the required evidence and prior approval by the FDA	**21 CFR 314.70** **21 CFR 601.12**
	**Approval Timelines**	Review timelines for supplemental applications are not specified by related regulations *Note: The Prescription Drug User Fee Act (PDUFA) has performance goals for supplemental efficacy and manufacturing changes as well to be 10 months from filing date of standard applications and 6 months for priority review applications *[113]	**21 CFR 314.70** **21 CFR 601.12** *21 USC 379g to 379h-2*
Facilitated Pathways or Tools used for Covid-19 MAs	**Emergency Use Authorization**	**Criteria and Conditions:** EUA is applicable for drugs, biologics, and devices The FDA issues the EUA subject to conditions based on following criteria: 1) if the medical product is for serious or life-threatening disease or condition, 2) if there is evidence of effectiveness, 3) if benefits outweigh the potential known risks, 4) if there are no alternatives **Procedure**: When the Secretary of the Department of Health and Human Services HHS declares that the emergency use authorization is appropriate, the FDA may authorize unapproved medicinal product or unapproved use of approved medicinal product for the emergency cases following EUA application by the sponsor. This declaration is based on one of the four types of determinations for threats or potential threats issued by the Secretary of HHS, Homeland Security, or Defense **Approval Timeline:** The EUA application review timeline by the FDA is not specified by the regulation. However, the FDA shall take actions to expedite the development and review of medicinal products for emergency uses *Note: FDA is prepared to issue EUA expeditiously (i.e., within hours or days) according to a guidance published by US FDA *[7] **Validity:** The EUA will stay effective until termination or revocation of the declaration by secretary when the circumstances for issuance of such authorization are no longer exist	**21 USC 360bbb-3** **21 USC 360bbb-3c**
	**Fast Track Designation**	**Criteria & Conditions:** The designation shall facilitate the development and expedite the review of the drug application if it is intended for a serious or life-threatening disease or condition treatment and if it addresses unmet medical needs, or if the Secretary designates the drug as a qualified infectious disease product under US Code Sect. 355f(d) **Procedure**: Fast track designation can be requested by the sponsor with or any time after submission of an application for the investigation of the drug. The FDA shall response to the request within 60 calendar days from the receipt of the request The regulation does not specify rolling review procedure associated with this designation *Note: A guideline published by FDA for expedited programs mentions additional rolling review feature for fast track designation *[6] **Approval Timeline:** The related regulation does not specify any review timeline **Validity:** NA	**21 USC 356(b)**
	**Priority Review**	**Criteria & Conditions:** Priority review voucher can be granted - If the drug is for serious condition and provides significant improvement in safety or effectiveness - If the drug is designated as a qualified infectious disease product - If the drug is intended for rare pediatric disease - If the drug is for treatment of agents that present national security threats **Procedure:** Priority Review voucher can be issued by the Secretary to the sponsor upon request (with original NDA, BLA or efficacy supplement) **Approval Timeline:** The priority review voucher shall ensure the review and action by the Secretary not later than 6 months after receipt by the Secretary of such application It is referenced also to Prescription Drug User Fee Act, which has performance goals for the review timelines to be 6 months from filing date for priority review NDAs, BLAs, or supplemental changes applications [113] **Validity:** NA	**21 USC 360bbb-4a** **21 USC 360n-1** **21 USC 360ff** **21 USC 379g to 379h-2**

By analyzing the key points of both EU and US regulations utilized in marketing authorization of Covid-19 vaccines and treatments, the following similarities and differences, and effectiveness of regulations were observed:

**Similarities:**

- Both EU and US regulations have provisions for MA of medicinal products that can be used during recognized public health threats like pandemics to achieve accelerated authorization. These are EU Conditional MA and US Emergency Use Authorization regulations.
- There are no stipulated approval timelines for either EU Conditional MA or US EUA. They can be granted as soon as the criteria and minimum requirements are fulfilled.
- The requirements related to clinical studies are similar. Both regulations specify the main characteristic of clinical studies to be adequate and well controlled without mentioning the number of required pivotal clinical studies.
- Rolling review features, in which the sponsor can submit data in many rolling review cycles as they become available before the formal application submission, are not mentioned in either EU or US regulations. Both regulatory authorities introduced this tool through guidelines, which are not legally binding.

**Differences:**

- In USA, drugs and biologics are regulated separately and there are different application types (NDA and BLA) for MA. In the EU, drugs and biologics are regulated under the same regulations with the same MA application.
- The EU conditional MA follows the same procedure as the standard MA, whereas the US EUA is a different procedure than the standard NDA or BLA.
- The EU has one centralized application, which results in either standard or conditional MA depending on the completeness of the submitted evidence. In USA, there is EUA application at the beginning and separate NDA/BLA at a later stage if the sponsor intends to obtain standard approval.
- The EU conditional MA has more strictly regulated post-authorization obligations, whereas US EUA is granted based on a sponsor’s plans for clinical trials continuation and mentions only identified gaps.
- The EU conditional MA regulation is applicable only to authorization of new entities but not for addition of new therapeutic indications for already-authorized entities. The US EUA regulation is applicable for both new entities and new indications of existing entities.
- The EU variation regulation allows authorization with missing pharmaceutical, clinical, or non-clinical data subject to post-authorization obligation in emergency cases such as a pandemic. US variations regulation does not mention anything about the acceptability of missing data.
- The EMA regulation stipulates that a conditional MA is renewed annually, whereas a US EUA stays valid until termination, or it is revoked by declaration by secretary if the circumstances for issuance of such authorization no longer exist.
- Unlike EU, the “rolling review” feature, in which the sponsor can submit data in many rolling review cycles as they become available before the formal application submission, is not a standalone tool in the US but is instead considered one of the features related to “Fast Track Designation” as per FDA guidance. The “Fast Track Designation” itself is regulated by US Code.

## Effectiveness of the Utilized Regulations:

**Figure 5:**
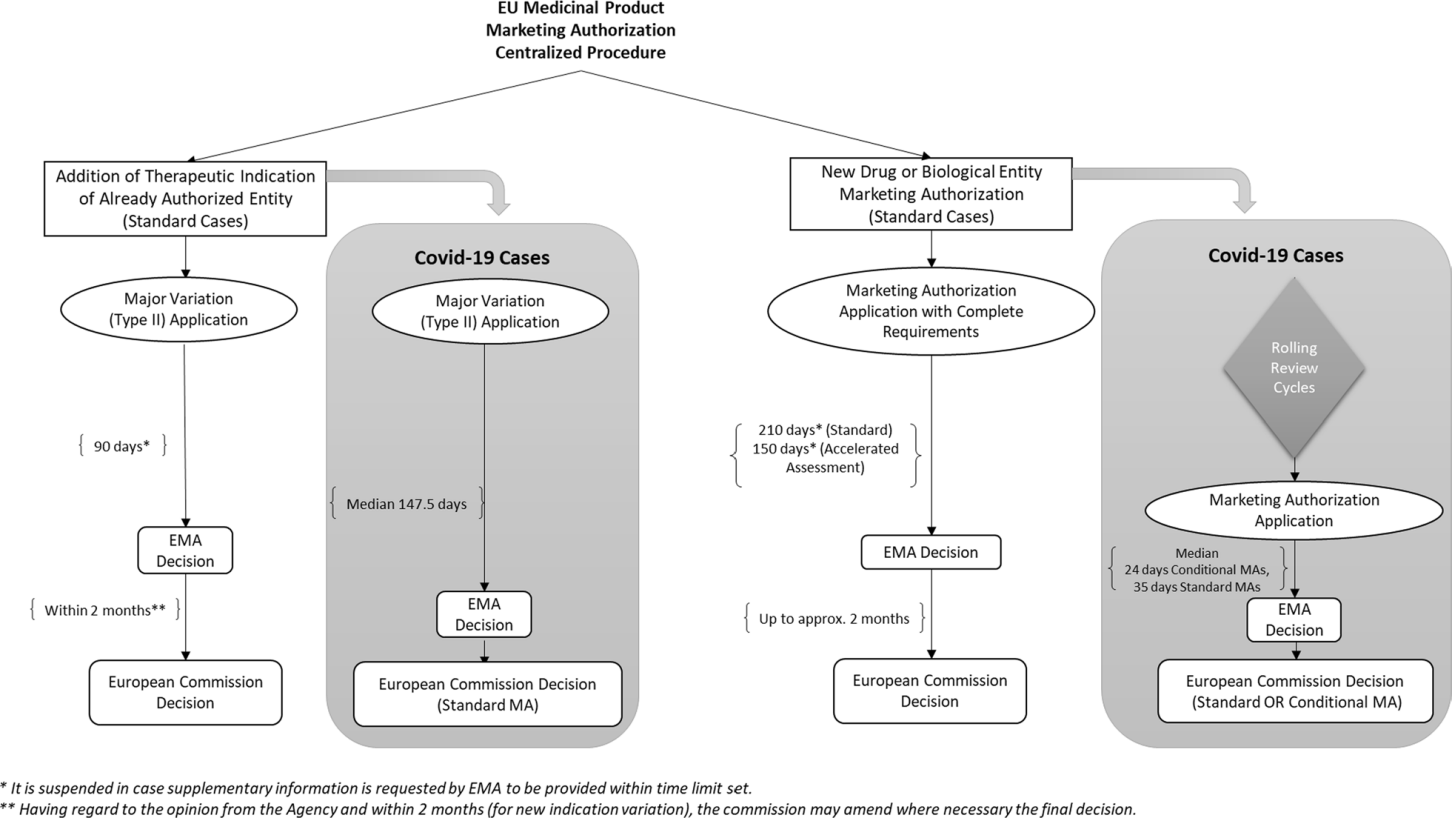
EU MA centralized procedure both for standard and implemented Covid-19 cases.

**Figure 6:**
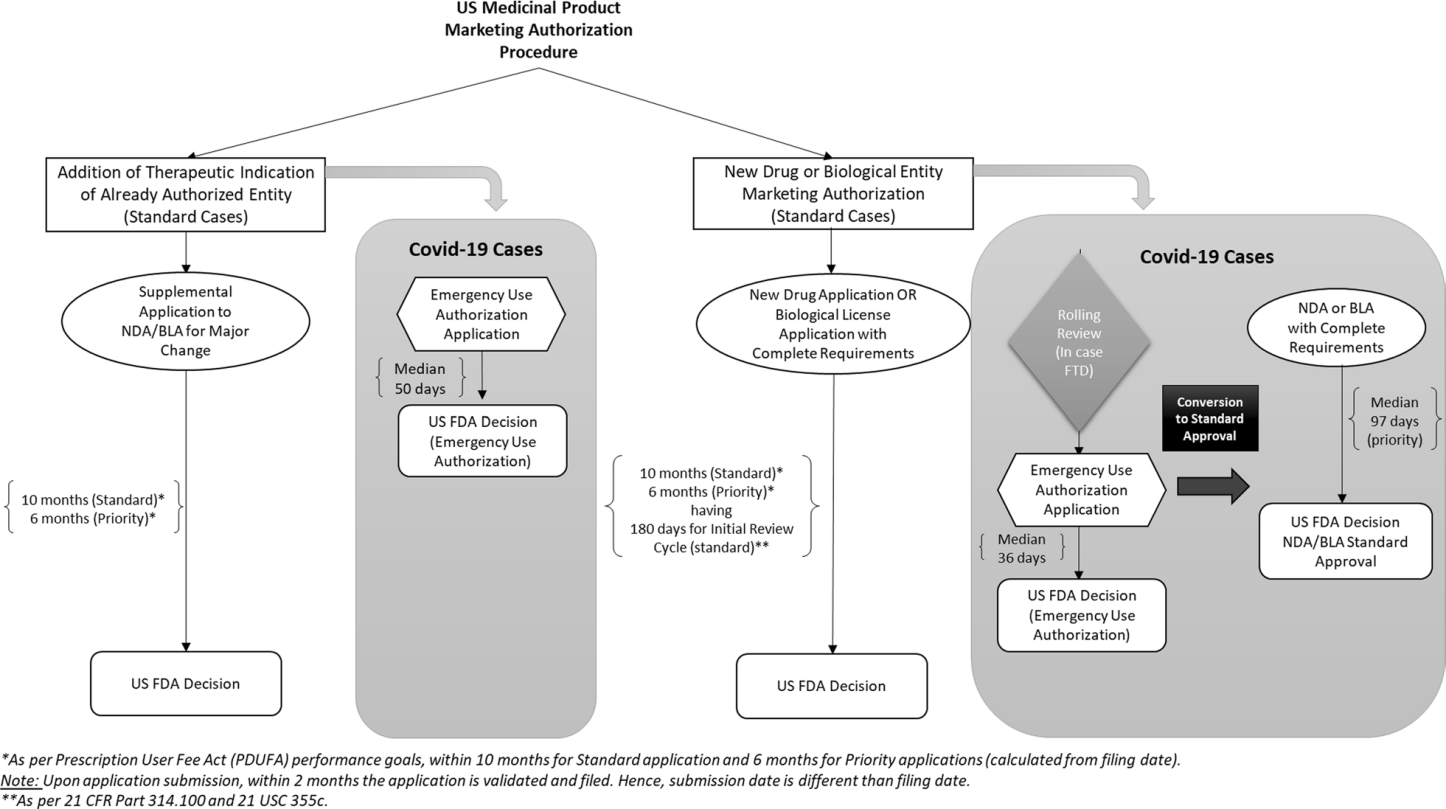
US Procedures both for standard and implemented Covid-19 cases.

By assessing the utilized regulations against the time and safety standards listed under Sect. "Identification and Comparison of the Utilized EU and US Regulations for Covid-19 MAs", the following is observed:

1. For most cases, Covid-19 MAs had faster approval timelines compared to the standard MAs. EU and US timelines and procedures implemented for COVID-19 case, and EU and US timelines and procedures used for other standard case approvals are illustrated in Figs. 5 and 6, respectively.
2. The EU conditional and US EUA regulations were flexible. Additional facilitated pathways were implemented to further reduce the review timelines. At the same time, they both mandated strict criteria and conditions to be met as listed in Tables 12 and 13, respectively. Also, they enabled regulatory authorities to issue obligations to instruct continuation of the studies for more comprehensive data.
3. The clinical trials with safety evidence, which supported the Covid-19 MAs, were all in line with the current regulations and requirements.
4. Despite the fast approval timelines, the regulatory authorities were able to perform comprehensive reviews of the supporting evidence well in advance of the application submission through the rolling review feature.

There was an observation for visibility of such details in US initial EUAs. This is converted to a point for enhancement as recommendation to regulators under section "Recommendations to Regulators".

## Discussion

Prompted by the Covid-19 pandemic, this study aimed to compare and contrast EU and US regulations, processes, and outcomes of granting accelerated marketing authorizations for Covid-19 vaccines and treatments, in order to determine how effective these regulations were in delivering safe medicines in a timely fashion during an emergency situation. All accelerated MAs for Covid-19 vaccines and treatments, and the associated utilized regulations, were identified and compared between the EU and USA, which enabled the identification of both efficiencies and limitations in regulations. Accordingly, recommendations are made for future enhancement.

### Historic and Current Uses of Facilitated Pathways

Regulations for accelerated review of MA applications for new medicines have been developed in the EU and USA over the past two decades, so as to accelerate access to medicines treating serious conditions or unmet need. Many facilitated pathways exist in the US, which benefited the majority of new molecular entities in USA [24, 25, 27]. Facilitated pathways have also been used in the EU, but long administrative periods between EMA’s decision and final European Commission’s approval limited accelerated access [33, 34]. However, these historic delays in the EU were not observed during the Covid-19 pandemic.

Many studies investigating regulatory authority application review approval timelines for new innovative medicines reported that USFDA had faster review timelines and that new drugs were first approved in USA [15, 16, 43, 44]. This study showed that this continued during Covid-19. However, this was not because of faster approval USFDA timelines but because sponsors first submitted MA applications in USA and later in the EU.

Granting fast approvals for new medicines for serious conditions or unmet medical needs is, of course, not a new concept applied during the Covid-19 pandemic. Nevertheless, the Covid-19 pandemic emphasized the importance of good communication strategies which address public concerns and hesitancies which emerged in relation to the speed of Covid-19 vaccines and treatments regulatory approvals [17–19].

### EU and US Regulations Utilized for Covid-19 MAs

The EU conditional MA and US emergency use authorization procedures had been introduced to facilitate accelerated MA of safe and effective new medicines during public health threats such as duly recognized pandemics. While the relevant regulations did not specify any review timelines, they were accompanied with other facilitated tools to further accelerate the development and review timelines.

This study has shown that the US and EU regulations both specify the main characteristics of clinical trials evidence for safety and efficacy, and that these need to be adequate and well controlled. The regulations in the EU and USA do not, however, mention how many pivotal clinical studies are required [45], and the standard perceived minimum of two pivotal clinical trials requirement in USA is based on a USFDA guidance document [42], without legally enforceable responsibilities. Hence, Covid-19 MAs based on one pivotal clinical trial was in line with current requirements.

Downing et al. [46] found that the clinical trials supporting both standard and facilitated US approvals for all novel drugs between 2005 and 2012 were randomized, double-blinded, and used either an active or placebo comparator. These authors also found that the median number of pivotal trials per indication was two both for standard and facilitated approvals, although 36.8% were approved based on a single pivotal trial.

Morant and Vestergaard [47] investigated whether there were differences in the reported number of EU pivotal clinical trials for standard and facilitated approvals. They found that between 2012 and 2016, indications approved via standard procedure were mostly based on clinical evidence from two or more pivotal clinical trials (61%), while the majority of the indications with a conditional MA (85%) and indications with a MA under exceptional circumstances (80%) were based on a single pivotal clinical trial.

Leyens et al. [48] found that between 2007 and 2015, there were differences in the accepted clinical trials characteristics supporting the facilitated approvals of new medicines between USFDA and EMA. For example, all EMA approvals were based at least on clinical phase II data, whereas the USFDA granted two approvals (out of 25) based on only phase I data. Indeed, most USFDA approvals were based on one or two clinical trials, whereas EMA approvals were based on at least two. However, the USFDA more often requested new confirmatory randomized controlled clinical trials, whereas EMA's common request was the completion of ongoing trials.

The current study revealed that both EU and US regulations were sufficiently effective to ensure fast authorization of Covid-19 vaccines and treatments during the pandemic.

During the Covid-19 pandemic, the EU conditional MA accompanied by a rolling review procedure proved its worth for new entities. New indication approval of already-authorized entities did not, however, benefit from EU conditional MA and rolling review, so did not have accelerated authorization. In USA, the regulations of emergency use authorization were applicable to both new entities and new indications and hence both had faster approvals.

By studying the relevant regulations, the present study found that both US and EU regulations do not have provisions for rolling review, but that both authorities introduced this tool through non-legally binding guidelines. In the EU, the rolling review procedure was used as a standalone procedure under the EMA’s initiatives for acceleration of development support and evaluation procedures for Covid-19 treatments and vaccines [49]. In USA, rolling review is one of the features related to fast track designation.

This study showed that EU and US procedures were effective in ensuring that medicines approved with accelerated authorization were safe and effective. The rolling review ensured that dossier content and all data were reviewed well in advance of the official application submission and both conditional MA and emergency use authorization regulations specify criteria and conditions for authorization.

### Importance of EMA and USFDA Approvals for Covid-19 Medicines

The WHO declared Covid-19 a global pandemic in March 2020 [1], and the USFDA and EMA initiatives to accelerate the development of promising vaccines and treatments and grant fast approvals thus had high importance during this time. Two years in, in March 2022, there were over 500 million confirmed Covid-19 cases worldwide, and the pandemic had claimed around 6 million lives [50].

The off-label use of medicines, whereby medicines are used for unapproved indications, increased during the Covid-19 pandemic [51]. Hence, it was crucial for regulatory authorities to grant speedy, official authorization to Covid-19 medicines to ensure safe and effective treatments. The USFDA was the fastest to grant an initial official emergency use authorization in May 2020 for Remdesivir, for the treatment of hospitalized patients with severe Covid-19 conditions. The authorization of Covid-19 convalescent plasma followed in August 2020, with all other US and EU approvals being granted gradually from November 2020.

### Study Limitations and Strengths

One limitation of the current study is that it examined the Covid-19 MAs granted by two regulatory authorities only. However, the USFDA and EMA are two of the largest regulatory authorities, and the importance of their approvals is not limited to the USA and EU. They have big worldwide impact since many regulatory authorities rely on USFDA and/or EMA approvals to authorize medicines in their regions [52–54]. Accelerated and emergency approvals by USFDA and EMA had a major impact on saving millions of lives and on slowing down the progression of the pandemic, which had global economic effects [55].

Because the USFDA only publishes the full review history with the final NDA/BLA standard approval and not with emergency use initial authorization letter, the current study was unable to confirm if there were additional facilitated pathways beside the emergency use authorization for US Covid-19 vaccines and treatments, which were not converted to standard approval. The study did not search in press releases to avoid bias as some sponsors might not publish these details. Instead, the publication of the review history of facilitated pathways together with the emergency use initial review memorandums is picked up as one recommendation to regulators.

Another limitation is that the study did not address any revoked or terminated MAs in the study period. However, this is not due to limitation in information sources but because including revoked MAs will not provide efficient comparison in the outcomes and will not add value in addressing the aim of the study.

The current study also has many strengths. It investigated the facilitated pathways for marketing authorizations of new medicines introduced by two of the largest regulatory authorities worldwide. It provides an overview of the use of facilitated pathways historically and investigated their special use for Covid-19 vaccines and treatments. It is the first study, which compared the US emergency use authorization and EU conditional MA pathways, which are the only legal tools available for use during recognized pandemics. Moreover, this study investigated both new medicines and the addition of new therapeutic indication to already-authorized medicines. Furthermore, besides investigating Covid-19 MAs, this study also explored the underpinning regulations, which allowed a comparison of procedures used for standard vs. Covid-19 cases.

### Recommendations to Regulators

Based on this study, the below points are recommendations for regulators in order to enhance regulation efficiency, especially during an emergency situation.

1. Addition of provisions related to conditional variation approval for therapeutic indication addition to the European Committee Regulation (EC) No 507/2006 related to conditional marketing authorization.
2. Addition of review history related to additional expedited pathways into the US published review memorandum of the initial emergency use authorization. This will ensure transparency of evidence reviewed by USFDA under expedited pathway like fast track designation and by rolling review prior to official application submission.
3. Addition of provisions related to rolling review procedure to both the EU and US regulations, so that the sponsor can submit data as they become available in many rolling review cycles and before the formal application submission. These provisions can then standardize the rolling review process, conditions, criteria, and timelines.

## Conclusion

Despite some variability and minor limitations found in regulations, this research demonstrated the overall efficiency of both US and EU regulations and practice in ensuring accelerated market access to safe and effective Covid-19 medicines during the pandemic. It opposed the perceived historic view that the USFDA is always faster than EMA in ensuring market access to new innovative medicines. In fact, the first US Covid-19 approvals were not related to faster approval timelines or more efficient practice, because the majority of sponsors submitted applications first in USA and later in EU. Provided sponsors submit in parallel in both regions, the populations in the USA and EU will receive parallel access to life saving medicines during any pandemic situation.

This study has further shown that fast approvals and accepted clinical trials were in alignment with current regulations and were not compromising approval standards related to safety or efficacy. Hence, communication strategies are suggested to also address public concerns and hesitancies toward Covid-19 vaccines and treatments with fast regulatory approvals.

### Supplementary Information

Below is the link to the electronic supplementary material.

Supplementary file1 (DOCX 736 KB)

## Data Availability

The analyzed data supporting the results reported in this article can be found through hyperlinks to publicly archived datasets.

## Abbreviations

BLA: Biological License Application
CFR: Code of Federal Regulations
CHMP: Committee for Medicinal Products for Human Use
EC: European Commission
EMA: European Medicines Agency
EPAR: European Public Assessment Report
EU: European Union
EUA: Emergency Use Authorization
IND: Investigational New Drug
MA: Marketing Authorization
MAH: Marketing Authorization Holder
NDA: New Drug Application
NME: New molecular entity
PRIME: Priority medicines scheme
US: United States
USC: United States Code
USFDA: United States Food and Drug Administration

## Footnotes

1 According to the Article 13(3) of Regulation (EC) No 726/2004 [31], EPAR is published for every human or veterinary medicine application that has been granted or refused a marketing authorization following assessment by Committee for Medicinal Products for Human Use or Committee for Medicinal Products for Veterinary Use of EMA in the framework of the Central authorization of medicines. It provides public information on a medicine, including how it was assessed by EMA reflecting the scientific conclusions and the history of the assessment [37].

2 US review memorandums were published to provide all the emergency use authorization application details, assessment history, product information, related regulatory submissions, and the summary of clinical data.

3 Eudralex is a collection of rules and regulations which govern medicinal products in European Union. It comprises of many volumes including legislations for veterinary use and other volumes related to all the guidelines [41].

4 The Code of Federal Regulations (CFR) is the codification of the general and permanent rules published in the Federal Register by the departments and agencies of the Federal Government. While the United States Code is a consolidation and codification by subject matter of the general and permanent laws of the United States. It is prepared by the Office of the Law Revision Counsel of the United States House of Representatives.
