# Supplementary material for: Comparison between European Medicines Agency and US Food and Drug Administration in Granting Accelerated Marketing Authorizations for Covid-19 Medicines and their Utilized Regulations
Source: Ther Innov Regul Sci. 2023 Oct 20;58(1):79–113. doi: 10.1007/s43441-023-00574-6 (PMC10764419; doi:10.1007/s43441-023-00574-6)
Supplement: Supplementary file 1 — Supplementary file1 (DOCX 736 KB) [file 43441_2023_574_MOESM1_ESM.docx]

# **TABLES**

****Table 1: USFDA Expedited Programs for New Drugs Approvals****

| Expedited Program | Fast Track Designation | Breakthrough Therapy Designation | Accelerated Approval | Priority Review |
| --- | --- | --- | --- | --- |
| Eligibility Criteria | Drug is for serious condition  AND  pre-clinical or clinical data demonstrates potential to address unmet need  OR  The drug is designated as a qualified infectious  disease product | Drug is for serious condition  AND  its preliminary clinical evidence indicates  substantial  improvement on a  clinically significant  endpoint(s) over  available therapies | Drug is for serious condition  AND  has advantage over available therapies  AND  Demonstrates an effect on a surrogate endpoint that predicts clinical benefit or clinical endpoint that can be measured earlier than irreversible morbidity or mortality. | Drug is for serious condition  AND  Provides significant improvement in safety or effectiveness  OR  The drug is designated as a  qualified infectious disease product  OR  Any supplement for  labeling change to a report on a pediatric study under 505Ab  OR  Any application or supplement submitted with a priority review voucher. |
| Requesting Time | With or after IND  No later than pre-NDA or pre-BLA meeting | With or after IND  No later than end of phase 2 meeting | Possibility of accelerated approval is discussed with review division during development | With original NDA, BLA or efficacy supplement |
| Expediting Features | Actions to expedite development and approval  Rolling review* | Actions to expedite review  Rolling review*  Guidance on  efficient development  Organizational  commitment | Approval based on surrogate endpoint or intermediate clinical endpoint likely to predict a drug’s clinical benefit. | Shorter review of  Marketing application (6  months compared with the 10-month standard review as per PDUFA goals) |

** Rolling Review means that a drug company can submit completed sections of its BLA or NDA for review by FDA, rather than waiting until every section is completed before the entire application can be reviewed* ***[56]****.*

Table 2: EMA Facilitated Pathways for New Marketing Authorizations

| Facilitated Pathway | Conditional MA | MA under Exceptional Circumstances | Accelerated Assessment | Priority Medicines (PRIME) Scheme |
| --- | --- | --- | --- | --- |
| Eligibility Criteria | If comprehensive safety and efficacy clinical data is not provided, but if the applicant is able to provide comprehensive data in future.  For medicinal products for seriously debilitating or life-threatening diseases,  or  for medicinal products needed in emergency situations,  or  in response to public health threats duly recognized either by the World Health Organization or by the Community in the framework of Decision No 2119/98/EC;  or  for orphan designated medicinal products for rare diseases. | If comprehensive data cannot be provided and the applicant can justify, authorization can be granted under specific procedures in exceptional circumstances for objective verifiable reasons. | If the medicinal product has interest from a public health point of view and if it demonstrates therapeutic innovation. | If the medicinal product has major public health interest from therapeutic innovation point of view  and  it is for unmet medical need or if it has advantage over other existing therapies. |
| Requesting Time | At the time of application for marketing authorization. *(It may be requested by the applicant or proposed by the CHMP).* | In advance of the Marketing Authorization application. | 2-3 months before the actual submission of the marketing authorization application in order to allow sufficient time for its assessment. | Early stages of development; from exploratory clinical trial phase with proof of concept, or in exceptional cases at an earlier stage of development. |
| Expediting Features | Authorization based on the obligations to provide comprehensive data in future. However, it is valid for one year subject for renewal and assessment of specific obligations. | Authorization without comprehensive data. However, it is subject to annual assessment of the conditions and justifications. | Application review timeline is reduced to 150 days instead of the standard 210 days. | Support provided for the development of the medicinal product by enhancing the interaction and early dialogue with the developers and providing scientific advice. |

****Table 3: USFDA and EMA Equivalent Applications and Pathways****

|  | | US Food and Drug Administration | European Medicines Agency |
| --- | --- | --- | --- |
| Marketing Authorization Approvals – Standard Applications | | New Drug Application (NDA)  Biological License Application (BLA) | Marketing Authorization Application |
| Marketing Authorization Approvals – “Facilitated Pathways” * | **Promoting Clinical Development** | Breakthrough Therapy Designation | Priority Medicines (PRIME) Scheme |
|  | **Reducing MA review timeline** | Fast Track Designation  Priority Review | Accelerated Assessment |
|  | **MAs without comprehensive clinical studies** | NA | MA Under Exceptional Circumstances |
|  | **MAs prior clinical confirmatory studies** | Accelerated Approval | Conditional Marketing Authorization |
|  | **MAs during recognized emergency public health threats** | Emergency Use Authorization |  |

*** *“Facilitated Pathway” term is used to collectively describe all the available expedited, accelerated, emergency use and conditional pathways.*

Table 4: Inclusion and Exclusion Criteria for Covid-19 Vaccines and Treatments MAs

| **Inclusion Criteria** | **Exclusion Criteria** |
| --- | --- |
| - Initial USFDA and centralized EMA official authorizations for Covid-19 vaccines and treatments by either standard marketing authorization or any of the facilitated pathways like US emergency use authorization or EU conditional marketing authorization. - Covid-19 vaccines and treatments including drugs and non-vaccine biological products which contain new entities i.e., new active chemical or biological substances. - Covid-19 treatment new indication authorizations for products which contain previously authorized entities. | - Investigational new drugs which are being tested in clinical trials for Covid-19 and/or submitted for marketing authorization application but have not yet received official marketing authorization from USFDA or EMA. - Covid-19 vaccines and treatments that are under research and development and have received EMA advice or under evaluation / rolling review or review under Article 5(3) of the Regulation (EC) No 726/2004 by EMA but not yet received official authorization. - Authorized medical devices related to Covid-19 such as in-vitro diagnostic test kits, protective equipment, ventilators, etc. - Emergency use authorizations of products to be used during the Covid-19 pandemic but not indicated for Covid-19 treatment (e.g., general anesthetics or replacement solutions). - Any revoked Covid-19 vaccine or treatment authorizations during the study period. |

Table 5: Inclusion and Exclusion Criteria for EU and US Utilized Regulations

| **Inclusion Criteria** | **Exclusion Criteria** |
| --- | --- |
| Legislation related to:   - Marketing authorizations of medicines for human use. - Marketing authorizations of new drugs and biologics. - Variation approval for new indication addition of previously authorized entities. - Facilitated pathways used in marketing authorizations of Covid-19 vaccines and treatments. - Only procedures related to marketing authorization. | Legislation related to:   - Marketing authorizations of medicines for veterinary use. - MAs of other product types not related to Covid-19 treatments and vaccines e.g., medical devices, herbal, orphan medicines, food, cosmetics, etc. - Other lifecycle management activities of medicines like registration renewals. - Facilitated pathways not used in marketing authorizations of Covid-19 vaccines and treatments. - All other procedures such as conducting clinical trials, clinical trials applications / INDs, medicines manufacturing, distribution, advertising, pharmacovigilance, pricing, etc. |

Table 6: Covid-19 Vaccines and Treatments authorized by EMA and USFDA by 28 Feb 2022

| **Entity / Product Name** | **Product Category** | **Entity Status** | **Sponsor in USA /**  **MAH in Europe** | **References** |
| --- | --- | --- | --- | --- |
| **Covid-19 Vaccines / Treatments authorized by both USFDA and EMA** | | | | |
| **Pfizer- BioNTech COVID-19 Vaccine /** [Comirnaty] | Vaccine | New Entity | USA: Pfizer, on behalf of Pfizer and BioNTech)  Europe: BioNTech Manufacturing GmbH | USFDA Authorization Letter / Review memo. **[57, 58]**  EMA EPAR **[59]** |
| **Janssen COVID-19 Vaccine** | Vaccine | New Entity | USA: Janssen Biotech, Inc.  Europe: Janssen-Cilag International NV | USFDA Authorization Letter / Review memo. **[60, 61]**  EMA EPAR **[62]** |
| **Moderna COVID-19 Vaccine /**  [Spikevax] | Vaccine | New Entity | USA: Moderna TX  Europe: Moderna Biotech Spain S.L. | USFDA Authorization Letter / Review memo. **[63, 64]**  EMA EPAR **[65]** |
| **Casirivimab co-packaged with Imdevimab /**  [Ronapreve in EMA,  REGEN-COV in USFDA] | Treatment | New Entity | USA: Regeneron Pharmaceuticals, Inc.  Europe: Roche Registration GmbH | USFDA Authorization Letter / Review memo. **[66, 67]**  EMA EPAR **[68]** |
| **Remdesivir /**  [Veklury] | Treatment | New Entity | USA: Gilead Sciences, Inc.  Europe: Gilead Sciences Ireland UC | USFDA Authorization Letter / Review memo. **[69, 70]**  EMA EPAR **[71]** |
| **Sotrovimab /**  [Xevudy] | Treatment | New Entity | USA: Pfizer, Inc.’s (Pfizer)  Europe: Pfizer Europe MA EEIG. | USFDA Authorization Letter / Review memo. **[72, 73]**  EMA EPAR **[74]** |
| **Nirmatrelvir co-packaged with ritonavir /** [Paxlovid] | Treatment | New Entity | USA: GlaxoSmithKline Research & Development Limited  Europe: GlaxoSmithKline | USFDA Authorization Letter / Review memo. **[75, 76]**  EMA EPAR **[77]** |
| **Tocilizumab /**  [RoActemra in EMA,  Actemra is USFDA] | Treatment | New indication of previously authorized entity | USA: Genentech, Inc.’s (Genentech)  Europe: Roche Registration GmbH | USFDA Authorization Letter / Review memo. **[78, 79]**  EMA EPAR **[80]** |
| **Covid-19 Vaccines / Treatments authorized by EMA only** | | | | |
| **AstraZeneca COVID-19 Vaccine /** [Vaxzevria] | Vaccine | New Entity | Europe: AstraZeneca AB | EMA EPAR **[81]** |
| **COVID-19 Vaccine (recombinant, adjuvanted) /**  [Nuvaxovid] | Vaccine | New Entity | Europe: Novavax CZ, a.s. | EMA EPAR **[82]** |
| **Aanakinra /**  [Kineret] | Treatment | New indication of previously authorized entity | Europe: Swedish Orphan Biovitrum AB (publ) | EMA EPAR **[83]** |
| **Regdanvimab /**  [Regkinora] | Treatment | New Entity | Europe: Celltrion Healthcare Hungary Kft | EMA EPAR **[84]** |
| **Covid-19 Vaccines / Treatments authorized by US FDA only** | | | | |
| **Molnupiravir** | Treatment | New Entity | USA: Merck Sharp & Dohme Corp.’s (Merck) | USFDA Authorization Letter / Review memo. **[85, 86]** |
| **Tixagevimab co-packaged with Cilgavimab /**  [Evusheld] | Treatment | New Entity | USA: AstraZeneca Pharmaceuticals LP’s (AstraZeneca) | USFDA Authorization Letter / Review memo. **[87, 88]** |
| **Bamlanivimab + Etesevimab** | Treatment | New Entity | USA: Eli Lilly and Company | USFDA Authorization Letter / Review memo. **[89, 90]** |
| **Baricitinib /**  **[**Olumiant] | Treatment | New indication of previously authorized entity | USA: Eli Lilly and Company | USFDA Authorization Letter / Review memo. **[91, 92]** |
| **COVID-19 convalescent plasma** | Treatment | New Entity* | USA: The Office of the Assistant Secretary for Preparedness and Response (ASPR)  U.S. Department of Health and Human Services (HHS) | USFDA Authorization Letter / Review memo. **[93, 94]** |
| **Bebtelovimab** | Treatment | New Entity | USA: Eli Lilly and Company | USFDA Authorization Letter / Review memo. **[95, 96]** |

**The content is not new chemical or biological entity itself. However, the product was not available previously in any form and for any other condition.*

Table 7: Comparison between EMA and USFDA in Covid-19 MAs Approval Timelines and Pathways

|  | | **European Medicines Agency** | | | | | **US Food and Drug Administration** | | | | |
| --- | --- | --- | --- | --- | --- | --- | --- | --- | --- | --- | --- |
| **Entity / Common Name(s)** | **Entity Status / Product Type** | **Approval Pathway(s)** | **Submission Date** | **Approval Date** | **Approval Timeline**  **(days)** | **Conversion to Standard Approval** | **Approval Pathway(s)** | **Submission Date** | **Approval Date** | **Approval Timeline**  **(days)** | **Conversion to Standard Approval** |
| **Authorized both by EMA and USFDA** | | | | | | | | | | | |
| **Pfizer- BioNTech COVID-19 Vaccine /** [Comirnaty] | New Entity  Vaccine | Conditional MA  Rolling Review | 30-Nov-20 | 21-Dec-20 | 21 | **No** | Emergency Use Authorization  Fast Track Designation | 20-Nov-20 | 11-Dec-20 | 21 | **Converted to Standard Approval** |
| **Janssen COVID-19 Vaccine** | New Entity  Vaccine | Conditional MA  Rolling Review | 15-Feb-21 | 11-Mar-21 | 24 | **No** | Emergency Use Authorization | 04-Feb-21 | 27-Feb-21 | 23 | **No** |
| **Moderna COVID-19 Vaccine /**  [Spikevax] | New Entity  Vaccine | Conditional MA  Rolling Review | 30-Nov-20 | 06-Jan-21 | 37 | **No** | Emergency Use Authorization  Fast Track Designation | 30-Nov-20 | 18-Dec-20 | 18 | **Converted to Standard Approval** |
| **Casirivimab co-packaged with Imdevimab /**  [Ronapreve in EMA,  REGEN-COV in USFDA] | New Entity  Treatment | **Standard MA**  Rolling Review | 08-Oct-21 | 12-Nov-21 | 35 | **Already approved by standard pathway** | Emergency Use Authorization | 08-Oct-20 | 21-Nov-20 | 44 | **No** |
| **Remdesivir /**  [Veklury] | New Entity  Treatment | Conditional MA  Rolling Review | 05-Jun-20 | 03-Jul-20 | 28 | **No** | Emergency Use Authorization  Fast Track Designation | 16-Apr-2020 | 01-May-20 | 15 | **Converted to Standard Approval** |
| **Sotrovimab /**  [Xevudy] | New Entity  Treatment | **Standard MA**  Rolling Review | 17-Nov-21 | 17-Dec-21 | 30 | **Already approved by standard pathway** | Emergency Use Authorization | 24-Mar-2021 | 26-May-21 | 63 | **No** |
| **Nirmatrelvir co-packaged with ritonavir /** [Paxlovid] | New Entity  Treatment | Conditional MA  Rolling Review | 7-Jan-2022 | 28-Jan-2022 | 21 | **No** | Emergency Use Authorization  Fast Track Designation | 16-Nov-21 | 22-Dec-21 | 36 | **No** |
| **Tocilizumab /**  [RoActemra in EMA,  Actemra is USFDA] | New Indication  Treatment | **Standard Variation Approval** | 27-Jul-21 | 07-Dec-21 | 133 | **Already approved by standard pathway** | Emergency Use Authorization | 20-Apr-21 | 24-Jun-21 | 65 | **No** |
| **Authorized only by EMA** | | | | | | | | | | | |
| **AstraZeneca COVID-19 Vaccine /** [Vaxzevria] | New Entity  Vaccine | Conditional MA  Rolling Review | 11-Jan-21 | 29-Jan-21 | 18 | **No** | **NA** | | | | |
| **COVID-19 Vaccine (recombinant, adjuvanted) /**  [Nuvaxovid] | New Entity  Vaccine | Conditional MA  Rolling Review | 16-Nov-21 | 20-Dec-21 | 34 | **No** |  |  |  |  |  |
| **Aanakinra /**  [Kineret] | New Indication  Treatment | **Standard Variation Approval** | 08-Jul-21 | 17-Dec-21 | 162 | **Already approved by standard pathway** |  |  |  |  |  |
| **Regdanvimab /**  [Regkinora] | New Entity  Treatment | **Standard MA**  Rolling Review | 01-Oct-21 | 12-Nov-21 | 42 | **Already approved by standard pathway** |  |  |  |  |  |
| **Authorized only by USFDA** | | | | | | | | | | | |
| **Molnupiravir** | New Entity  Treatment | **NA** | | | | | Emergency Use Authorization | 08-Oct-21 | 23-Dec-21 | 76 | **No** |
| **Tixagevimab co-packaged with Cilgavimab /**  [Evusheld] | New Entity  Treatment |  |  |  |  |  | Emergency Use Authorization | 30-Sep-21 | 08-Dec-21 | 69 | **No** |
| **Bamlanivimab + Etesevimab** | New Entity  Treatment |  |  |  |  |  | Emergency Use Authorization | 16-Nov-20 | 09-Feb-21 | 85 | **No** |
| **Baricitinib /**  **[Olumiant]** | New Indication  Treatment |  |  |  |  |  | Emergency Use Authorization | 15-Oct-20 | 19-Nov-20 | 35 | **No** |
| **COVID-19 convalescent plasma** | New Entity  Treatment |  |  |  |  |  | Emergency Use Authorization | NA | 23-Aug-2020 | NA | **No** |
| **Bebtelovimab** | New Entity  Treatment |  |  |  |  |  | Emergency Use Authorization | 07-Jan-22 | 11-Feb-22 | 35 | **No** |

Table 8: Continued USFDA Approval Timelines and Pathways for Covid-19 MAs Converted to Standard Approval

| **Entity / Common Name(s)** | **Entity Status / Product Type** | **Application Type** | **Additional Facilitated Pathway(s)** | **Standard Application Submission Date** | **Standard Approval Date** | **Approval Timeline**  **(days)** | **Reference for NDA / BLA Summary Basis of Regulatory Action” / “Summary Review”** |
| --- | --- | --- | --- | --- | --- | --- | --- |
| **Pfizer- BioNTech COVID-19 Vaccine /** [Comirnaty] | New Entity  Vaccine | Biological License Application (BLA) | Priority Review for Clinical review | 18-May-21 | 23-Aug-21 | 97 | **[97]** |
| **Moderna COVID-19 Vaccine /**  [Spikevax] | New Entity  Vaccine | Biological License Application (BLA) | Priority Review | 24-Aug-21 | 31-Jan-22 | 160 | **[98]** |
| **Remdesivir /**  [Veklury] | New Entity  Treatment | New Drug Application (NDA) | Priority Review | 07-Aug-20 | 22-Oct-20 | 76 | **[99]** |

Table 9: Therapeutic Groups, Approved Indications, Accepted Pivotal Clinical Trials and Post Authorization Obligations for EMA and USFDA Covid-19 MAs

| **Region** | **Entity / Product Name** | **Pharmaco-therapeutic Group (WHO ATC Code)** | **Approved Initial Indications** | **Pivotal Phase 3 Clinical Studies Supporting the MA Application** | | | | **Post Authorization Clinical Obligations** |
| --- | --- | --- | --- | --- | --- | --- | --- | --- |
|  |  |  |  | **Number of Trials + (Study Code)** | **Characteristics** | **Planned Participants Number** | **Status** |  |
| **Authorized both by EMA and USFDA** | | | | | | | | |
| **EU** | **Pfizer- BioNTech COVID-19 Vaccine / [Comirnaty]** | J07BX03  “Vaccines, other viral vaccines” | Active immunisation to prevent COVID-19 caused by SARS-CoV-2 virus, in individuals 16 years of age and older. | 1  (C4591001) | Randomized, multinational, placebo-controlled, observer-blind | 44000 | Ongoing | Completion of ongoing trials *(as obligation for conditional authorization)* |
| **USA** |  |  |  |  |  |  |  | Completion of ongoing trials *(EUA based on sponsor’s plans for continuation) (c)* |
| **EU** | **Janssen COVID-19 Vaccine** | J07BX03  “Vaccines, other viral vaccines | Active immunisation to prevent COVID-19 caused by SARS-CoV-2 in individuals 18 years of age and older. | 1^(a)^  (VAC31518COV3001) | Randomized, placebo-controlled, double-blind | 40000 | Ongoing | Completion of ongoing trials *(as obligation for conditional authorization).* |
| **USA** |  |  |  |  |  |  |  | Completion of ongoing trials *(EUA based on sponsor’s plans for continuation) ^(c)^* |
| **EU** | **Moderna COVID-19 Vaccine /**  **[Spikevax]** | J07BX03  “Vaccines, other viral vaccines | Active immunisation to prevent COVID-19 caused by SARS-CoV-2 virus in individuals 18 years of age and older. | 1  (mRNA-1273-P301 | Randomized, stratified, placebo- controlled, observer-blind | 30000 | Ongoing | Completion of ongoing trials *(as obligation for conditional authorization)* |
| **USA** |  |  |  |  |  |  |  | Completion of ongoing trials *(EUA based on sponsor’s plans for continuation) ^(c)^* |
| **EU** | **Casirivimab co-packaged with Imdevimab /**  **[Ronapreve in EMA, REGEN-COV in USFDA]** | J06BD  “[Antiviral monoclonal antibodies](https://www.whocc.no/atc_ddd_index/?code=J06BD&showdescription=no)” | Treatment of COVID-19 in adults and adolescents aged 12 years and older weighing at least 40 kg who do not require supplemental oxygen and who are at increased risk of progressing to severe COVID-19  +  Prevention of COVID-19 in adult patients and in adolescent patients aged 12 years and older weighing at least 40 kg. | 1 (treatment indication)  (COV-2067)  +  1 (prevention indication)  (COV-2069) | Randomized, Placebo-controlled, Double-Blind | 4567  +  3029 | Completed | Not available  (Standard Authorization) |
| **USA** |  |  | Treatment of mild to moderate coronavirus disease 2019 (COVID-19) in adults and pediatric patients (12 years of age and older weighing at least 40 kg) with positive results of direct SARS-CoV-2 viral testing, and who are at high risk for progressing to severe COVID-19 and/or hospitalization. | 0  (Based on phase 1/2 trial)  (COV-2067) |  | 799 | Ongoing | Not available |
| **EU** | **Remdesivir /**  **[Veklury]** | J05AB16  “Antivirals for systemic use, direct acting antivirals” | Treatment of coronavirus disease 2019 (COVID 19) in adults and adolescents (aged 12 years and older with body weight at least 40 kg) with pneumonia requiring supplemental oxygen. | 1^(a)^  (CO-US-540-5776 / Protocol No. 20-0006) | Randomized, placebo-controlled, double-blind | 1063 | Ongoing | Completion of ongoing trials *(as obligation for conditional authorization.* |
| **USA** |  |  | Treatment of hospitalized patients with severe 2019 coronavirus disease (COVID-19). | 2^(a)^  (Protocol No.20-0006 /NCT04280705  +  Wang et al. (2020) Trial / NCT0425765) |  | 1063  +  237 | Ongoing  +  Terminated | Not available^(c)^ |
| **EU** | **Sotrovimab /**  **[Xevudy]** | J06BD  “[Antiviral monoclonal antibodies](https://www.whocc.no/atc_ddd_index/?code=J06BD&showdescription=no)” | Treatment of adults and adolescents (aged 12 years and over and weighing at least 40 kg) with coronavirus disease 2019 (COVID-19) who do not require oxygen supplementation and who are at increased risk of progressing to severe COVID-19. | 1^(a)^  (COMET-ICE) | Randomized, placebo-controlled, double-blinded | 1057 | Completed | Not available  (Standard Authorization) |
| **USA** |  |  | Treatment of mild-to-moderate  coronavirus disease 2019 (COVID-19) in adults and pediatric patients (12 years of  age and older weighing at least 40 kg) with positive results of direct SARS-CoV-2 viral testing, and who are at high risk for progression to severe COVID-19, including hospitalization or death. |  |  |  | Ongoing | Completion of ongoing Trials  *(As identified outstanding issues/data gaps)* |
| **EU** | **Nirmatrelvir co-packaged with ritonavir / [Paxlovid]** | “Antiviral for systemic use” | Treatment of coronavirus disease 2019 (COVID-19) in adults who do not require supplemental oxygen and who are at increased risk for progressing to severe COVID 19 | 1  (EPIC-HR, C4671005) | Randomized, placebo-controlled, double-blinded | 3100 | Completed | Additional confirmatory requirements / studies  *(As legally binding measures and recommendations)* |
| **USA** |  |  | Treatment of mild-to-moderate COVID-19 in adults and pediatric patients (12 years of age and older weighing at least 40 kg) with positive results of direct SARS-CoV-2 viral testing, and who are at high risk2for progression to severe COVID 19, including hospitalization or death. |  |  | 2246 | Ongoing | Additional confirmatory requirements / studies in addition to completion of ongoing trial  *(As identified outstanding issues/data gaps)* |
| **EU** | **Tocilizumab /**  **[RoActemra in EMA, Actemra is USFDA]** | L04AC07  “Immunosuppressants, Interleukin inhibitors” | Treatment of coronavirus disease 2019 in adults who are receiving systemic corticosteroids and require supplemental oxygen or mechanical ventilation. | 4  (COVACTA  +  EMPACTA  +  REMDACTA  +  RECOVERY) | 3 Randomized, placebo-controlled, double-blinded  1  randomized, controlled, open-label, platform trial (RECOVERY) | 452  +  388  +  649  +  4116 | Completed | Not available  (Standard Authorization) |
| **USA** |  |  | Treatment of coronavirus disease 2019 (COVID-19) in hospitalized adults and pediatric patients (2 years of age and older) who are receiving systemic corticosteroids and require supplemental oxygen, non-invasive or invasive mechanical ventilation, or extracorporeal membrane oxygenation (ECMO). |  |  |  |  | Not available |
| **Authorized only by EMA** | | | | | | | | |
| **EU** | **AstraZeneca COVID-19 Vaccine / [Vaxzevria]** | J07BX03  “Vaccines, other viral vaccines | Active immunisation to prevent COVID 19 caused by SARS-CoV-2, in individuals 18 years of age and older. | 2  (COV002  +  COV003) | Both  randomized, controlled, participant blind | 12390  +  10000 | Both Ongoing | Completion of ongoing trials *(as obligation for conditional authorization)* |
| **EU** | **COVID-19 Vaccine (recombinan, adjuvanted) /**  **[Nuvaxovid]** | J07BX03  “Vaccines, other viral vaccines | Active immunisation to prevent COVID-19 caused by SARS-CoV-2 in individuals 18 years of age and older. | 2  (2019nCoV-302  +  2019nCoV-301) | Both  randomized, controlled, observer blind | 15000  +  30000 | Both Ongoing | Not available  *(there is additional recommendation only for clinical efficacy )^(b)^* |
| **EU** | **Aanakinra /**  **[Kineret]** | L04AC03  “Immunosuppressants, Interleukin inhibitors” | Treatment of coronavirus disease 2019 (COVID-19) in adult patients with pneumonia requiring supplemental oxygen (low- or high-flow oxygen) who are at risk of progressing to severe respiratory failure determined by plasma concentration of soluble urokinase plasminogen activator receptor (suPAR) ≥ 6 ng/ml | 1  (SAVE-MORE) | Randomized, placebo-controlled, double-blind | 1060 | Completed | Not available  (Standard Authorization) |
| **EU** | **Regdanvima /**  **[Regkinora]** | J06BB  “[Specific immunoglobulins](https://www.whocc.no/atc_ddd_index/?code=J06BB&showdescription=no)” | Treatment of adults with coronavirus disease 2019 (COVID-19) who do not require supplemental oxygen and who are at increased risk of progressing to severe COVID-19. | 1  (CT-P59 3.2) | Randomized, placebo-controlled, double-blind | Part 1: 327  Part 2: 1315 | Completed | Not available  (Standard Authorization) |
| **Authorized only by USFDA** | | | | | | | | |
| **USA** | **Molnupiravir** | “Antiviral for systemic use” | Treatment of mild-to-moderate COVID-19 in adults who are at high-risk for progression to severe COVID-19, including hospitalization or death and  for whom alternative COVID-19 treatment options authorized by FDA are not accessible or clinically appropriate. | 1^(a)^  (MK-4482-002) | Randomized, placebo-controlled, double-blinded | 1433 | Completed | Additional confirmatory requirements / studies  *(As identified outstanding issues/data gaps)* |
| **USA** | **Tixagevimab co-packaged with Cilgavimab /**  **[Evusheld]** | J06BD03  “[Antiviral monoclonal antibodies](https://www.whocc.no/atc_ddd_index/?code=J06BD&showdescription=no)” | Pre-exposure prophylaxis of COVID-19 in adults and pediatric individuals (12 years of age and older weighing at least 40 kg):  • Who are not currently infected with SARS-CoV-2 and who have not had a  known recent exposure to an individual infected with SARS-CoV-2 **and**  • Who have moderate to severe immune compromise due to a medical condition or receipt of immunosuppressive medications or treatments **and** may not mount an adequate immune response to COVID-19 vaccination **or**  • For whom vaccination with any available COVID-19 vaccine, according to the approved or authorized schedule, is not recommended due to a history of severe adverse reaction (e.g., severe allergic reaction) to a COVID-19 vaccine(s) and/or COVID-19 vaccine component(s). | 1^(a)^  (PROVENT) | Randomized, placebo-controlled, double-blinded | 5197 | Ongoing | Additional confirmatory requirements / studies in addition to completion of ongoing trial  *(As identified outstanding issues/data gaps)* |
| **USA** | **Bamlanivimab + Etesevimab** | “[Antiviral monoclonal antibodies](https://www.whocc.no/atc_ddd_index/?code=J06BD&showdescription=no)” | Treatment of mild to moderate coronavirus disease 2019 (COVID-19) in adults and pediatric patients (12 years of age and older weighing at least 40 kg) with positive results of direct SARS-CoV-2 viral testing, and who are at high risk for progressing to severe COVID-19 and/or hospitalization. | 1^(a)^  (J2W-MC-PYAB / BLAZE-1) | Randomized, placebo-controlled, double-blinded | 3890 | Ongoing | Not available |
| **USA** | **Baricitinib /**  **[Olumiant]** | L04AA37  “[Selective immunosuppressants](https://www.whocc.no/atc_ddd_index/?code=L04AA&showdescription=no)” | Treatment of COVID-19 in hospitalized adults and pediatric patients 2 years of age or older requiring supplemental oxygen, non-invasive or invasive mechanical ventilation, or ECMO (in combination with remdesivir) | 1^(a)^  (Protocol No.  20-0006  (ACTT-2)  NCT04401579) | Randomized, placebo-controlled, double-blinded | 1033 | Completed | Not available |
| **USA** | **COVID-19 convalescent plasma** | “[Other](https://www.whocc.no/atc_ddd_index/?code=L04AA&showdescription=no) blood products” | Treatment of hospitalized patients with COVID-19. | Multiple small trials and criteria ^(d)^ | NA | NA | Completed | Additional confirmatory requirements / studies  *(As recommendation)* |
| **USA** | **Bebtelovimab** | [Antiviral monoclonal antibodies](https://www.whocc.no/atc_ddd_index/?code=J06BD&showdescription=no) | Treatment of mild-to-moderate  coronavirus disease 2019 (COVID-19) in adults and pediatric patients (12 years  of age and older weighing at least 40 kg) with positive results of direct SARS-CoV-2 viral testing, and who are at high risk for progression to severe COVID-19, including  hospitalization or death, and for whom alternative COVID-19 treatment options approved or authorized by FDA are not accessible or clinically appropriate. | 0  (Based on phase 1/2 trial)  PYAH; BLAZE-4  NCT04634409 | Randomized, placebo-controlled, double-blind, single dose  trial | 1416 | Ongoing | Additional confirmatory requirements / studies in addition to completion of ongoing trial  *(As identified outstanding issues/data gaps* |

1. *There are additional phase 3 / supportive clinical trials. However, they are not considered pivotal in the application to support the main approved indication and/or results are not provided.*
2. *Main obligations to complete post-authorization measures for conditional marketing authorization are related to quality of the product and not related to clinical trials.*
3. *US FDA requested new post marketing confirmatory studies. However, these requirements were issued after receiving BLA standard approval and not with initial emergency use authorization.*
4. *Authorized based on 1) historical evidence using convalescent plasma in prior outbreaks of respiratory viruses, 2) certain preclinical evidence; 3) results from small clinical trials and observational studies of convalescent plasma conducted during the current outbreak; and 4) data obtained from the ongoing National Expanded Access Treatment Protocol (EAP) sponsored by the Mayo Clinic.*

Table 10: Identified EU Legislations Utilized in Marketing Authorization of Covid-19 Vaccines and Treatments

| Identified EU Legislations |
| --- |
| - Directive 2001/83/EC of the European Parliament and of the Council of 6 November 2001 on the Community code relating to medicinal products for human use [30]. - Regulation (EC) No 726/2004 of the European Parliament and of the Council of 31 March 2004 laying down Community procedures for the authorisation and supervision of medicinal products for human and veterinary use and establishing a European Medicines Agency [31]. - Regulation (EC) No 1901/2006 of the European Parliament and of the Council of 12 December 2006 on medicinal products for paediatric use and amending Regulation (EEC) No 1768/92, Directive 2001/20/EC, Directive 2001/83/EC and Regulation (EC) No 726/2004 [100]. - Regulation (EC) No 1394/2007 of the European Parliament and of the Council of 13 November 2007 on advanced therapy medicinal products [101]. - Commission Regulation (EC) No 1234/2008 of 24 November 2008 concerning the examination of variations to the terms of marketing authorisations for medicinal products for human use and veterinary medicinal products [102]. - Commission Regulation (EC) No 507/2006 of 29 March 2006 on the conditional marketing authorisation for medicinal products for human use falling within the scope of Regulation (EC) No 726/2004 of the European Parliament and of the Council [32] - Commission Regulation (EC) No 1662/95 of 7 July 1995 laying down certain detailed arrangements for implementing the Community decision-making procedures in respect of marketing authorizations for products for human or veterinary use [103]. |

Table 11: Identified US Regulations Utilized in Marketing Authorization of Covid-19 Vaccines and Treatments

| Identified US Regulations |
| --- |
| - 21 CFR Part 314   Title 21 Food and Drugs > Chapter I Food and Drug Administration, Department of Health and Human Services  > Subchapter D Drugs for Human Use > Part 314 Applications for FDA Approval to Market a New Drug [104].   - 21 CFR Part 601   Title 21 Food and Drugs > Chapter I Food and Drug Administration, Department of Health and Human Services  > Subchapter F Biologics > Part 601 Licensing [23].   - 21 USC 355   Title 21 Food and Drugs > Chapter 9 Federal Food, Drug, And Cosmetic Act  > Subchapter V Drugs and Devices > Part A Drugs and Devices > Section 355 New Drugs [105].   - 42 USC 262   Title 42 The Public Health and Welfare > Chapter 6A Public Health Service  > Subchapter II General Powers And Duties > Part F Licensing Of Biological Products And Clinical Laboratories > Subpart 1 Bioloical product > Section 262 Regulation of Biological Products [106].   - 21 USC 356   Title 21 Food and Drugs > Chapter 9 Federal Food, Drug, And Cosmetic Act  > Subchapter V Drugs and Devices > Part A Drugs and Devices > Section 356 Expedited approval of drugs for serious or life-threatening diseases or conditions [107].   - 21 USC 356b   Title 21 Food and Drugs > Chapter 9 Federal Food, Drug, And Cosmetic Act  > Subchapter V Drugs and Devices > Part A Drugs and Devices > Section 356b Reports of post marketing studies [108].     - 21 USC 360bbb-3   Title 21 Food and Drugs > Chapter 9 Federal Food, Drug, And Cosmetic Act  > Subchapter V Drugs and Devices > Part E General Provisions Relating to Drugs and Devices > Section 360bbb-3 Authorization for medical products for use in emergencies [109].   - 21 USC 360bbb-3c   Title 21 Food and Drugs > Chapter 9 Federal Food, Drug, And Cosmetic Act  > Subchapter V Drugs and Devices > Part E General Provisions Relating to Drugs and Devices > Section 360bbb-3c Expedited development and review of medical products for emergency uses [110].   - 21 USC 360bbb-4a   Title 21 Food and Drugs > Chapter 9 Federal Food, Drug, And Cosmetic Act  > Subchapter V Drugs and Devices > Part E General Provisions Relating to Drugs and Devices > Section 360bbb-4a Priority review to encourage treatments for agents that present national security threats [111].   - 21 USC 379g to 379h-2   Title 21 Food and Drugs > Chapter 9 Federal Food, Drug, And Cosmetic Act  > Subchapter VII General Authority > Part C Fees > Subpart 2 Fees relating to drugs > Sections 379g to 379h-2 [112]. |

Table 12: Key Points from Identified EU Legislations

| **Search Area** | | **Finding** | **Reference Regulation** |
| --- | --- | --- | --- |
| **New Entities MAs** | **Procedure(s)** | There are four types of procedures used in EU for marketing authorization of medicinal products:   - Centralized Procedure - Decentralized Procedure - Mutual Recognition Procedure - National Procedure   **Procedure for Medicines intended for Viral Diseases:** Centralized procedure is the compulsory route for the medicinal products which contain active substances targeted for viral diseases. Hence, only centralized procedure is applicable for Covid-19 MAs, in which one MA application is submitted to the EMA. Upon approval, final authorization is issued by the European Commission, which will be valid for all EU member states. | **Directive 2001/83/EC**  **Regulation (EC) No 726/2004 (for Centralized Procedure)**  **Annex 1 - Regulation (EC) No 726/2004** |
|  | **Approval Timelines** | In standard cases, the EMA shall ensure the opinion of the Committee for Medicinal Products for Human Use is given within 210 days after receipt of a valid application in standard cases.  There are additional timelines for European Commission final decision-making procedure which take up to 52 days in case of approval decision.  *Note: In accelerated assessment procedure (not used for authorized Covid-19 vaccines and treatments), EMA shall ensure the opinion of the Committee for Medicinal Products for Human Use is given within 150 days instead of 210 days.* | **Article 6 of Regulation (EC) No 726/2004**  **Article 10 of Regulation (EC) No 726/2004,**  **Commission Regulation (EC) No 1662/95**  **Article 14(9) of Regulation (EC) No 726/2004** |
|  | **Clinical Trials Requirements** | Each application for medicinal product authorization shall specifically and completely include the particulars and documents referred in Directive 2001/83/EC.  Clinical trials shall be done as “controlled clinical trials”, randomized and as appropriate versus placebo and versus an established medicinal product of proven therapeutic value. Any other design shall be justified.  *Note: Reference regulation section specifying the number of required pivotal phase 3 clinical trials is not found.* | **Article 6 of Regulation (EC) No 726/2004**  **+**  **Directive 2001/83/EC** |
|  | **Post-Authorization Studies** | After granting marketing authorization, where necessary the EMA may impose obligations to the MAH to conduct post-authorization clinical safety and/or efficacy studies. | **Article 10(a) of Regulation (EC) No 726/2004** |
|  | **Other Notes** | For therapeutic indications intended to population under 18 years old, the provisions of the Regulation (EC) No 1901/2006 on medicinal products for paediatric use are applied.  For advanced therapy medicinal products., the provisions of the Regulation (EC) No 1394/2007 are applied. | **Regulation (EC) No 1901/2006**  **Regulation (EC) No 1394/2007** |
| **New Indication MAs of already Authorized Entities** | **Procedure** | New therapeutic indication addition is classified as major variation to the existing marketing authorization and requires type II variation application with all the required evidence and prior approval from the EMA and final decision from the European Commission.  ***Note:*** *Only where there is dully recognized pandemic situation with respect to human influenza or human coronavirus, the Commission may, where certain pharmaceutical, non-clinical or clinical data are missing, exceptionally and temporarily accept a variation to the terms of a marketing authorization and the MAH shall submit the missing data within a time limit set by the relevant authority.* | **Commission Regulation (EC) No 1234/2008**  **Article (21) of Commission Regulation (EC) No 1234/2008** |
|  | **Approval Timelines** | The EMA shall issue an opinion on the valid application within 90 days following its receipt in case of variations concerning a change to or addition of therapeutic indications. However, the agency may request supplementary information within a time limit set. In this case, the procedure shall be suspended until the supplementary information is provided.  Where the outcome of the assessment is favorable, the EMA shall transmit to the Commission its opinion within 15 days. The Commission, having regard to the opinion from the Agency and within 2 months (for new indication variation), shall amend where necessary the decision granting the marketing authorization.  The commission decision will be valid across all the EU member states. | **Articles 16, 17 and 23(a) of Commission Regulation (EC) No 1234/2008** |
| **Facilitated Pathways or Tools used for Covid-19 MAs** | **Conditional MA** | **Criteria & Conditions**:   - Conditional MA is applicable to medicinal products:   - - which aim at the treatment, the prevention or the medical diagnosis of seriously debilitating diseases or life-threatening diseases.     - to be used in emergency situations, in response to public health threats duly recognized either by the World Health Organization or by the Community in the framework of Decision No 2119/98/EC.     - designated as orphan medicinal products in accordance with Article 3 of Regulation (EC) No 141/2000. - Conditional MA can be granted based on less complete data than is normally the case and subject to specific obligations to complete the ongoing studies or conduct new studies. However, the following requirements should be met:   - - Risk benefit balance, as defined in Article 1(28a) of Directive 2001/83/EC should be positive.     - It is likely that the applicant will be in a position to provide the comprehensive clinical data.     - Unmet medical needs will be fulfilled.     - The benefit to public health of the immediate availability on the market of the medicinal product concerned outweighs the risk inherent in the fact that additional data are still required. - There is special note saying that where conditional MAs are granted, they should be restricted to situations where only the clinical part of the application dossier is less complete than normal. Incomplete pre-clinical or pharmaceutical data should be accepted only in the case of a product to be used in emergency situations, in response to public health threats.   **Procedure:**   - The procedure for evaluating a conditional marketing authorization is the normal procedure laid down in Regulation (EC) No 726/2004.   **Approval Timelines:**   - Conditional MA regulation does not specify any approval timelines.   **Validity:**   - Conditional MA will be valid for one year on a renewable basis. | **Commission Regulation (EC) No 507/2006** |
|  | **Rolling Review** | With Rolling Review process, the MAH can submit the data in many rolling review cycles as they become available from the ongoing studies to be reviewed by EMA before the formal application submission. Once the EMA’s human medicines committee decides that sufficient data are available, the MAH can submit the formal application.  This tool is introduced by EMA **[49]**. However, reference regulation section is not found. | **Not available** |

Table 13: Key Points from Identified US Regulations

| **Search Area** | | **Finding** | **Reference Regulation** |
| --- | --- | --- | --- |
| **New Entities MAs** | **Procedure(s)** | For new drugs, New Drug Application (NDA) should be submitted and approved by the US FDA before entering the market.  For new biological products including vaccines, Biological License Application (BLA) should be submitted and approved by the US FDA before entering the market.  Standard pathway is followed requiring full set of efficacy and safety reports for new molecular entities and original BLAs. This is referred also as Section 505(b)(1) standard application pathway as per Federal Food, Drug, And Cosmetic Act. | **21 CFR Part 314 +**  **21 USC 355**  **21 CFR Part 601 +**  **42 USC 262**  **21 USC 355 (b)(1) /**  **FFD&C Sec 505(b)(1) [21]** |
|  | **Approval Timelines** | In standard cases, FDA will review the New Drug Application within 180 days of the receipt “initial review cycle” and send back to the applicant either approval letter or complete response letter including the deficiencies. This timeline can be adjusted by mutual agreement between FDA and applicant.  In case of deficiencies and resubmission, there is additional review cycle of 2 months for class 1 minor resubmissions or 6 months for class 2 major resubmissions  The regulations for licensing biological products do not specify BLA review timelines.  *Note: The* *Prescription Drug User Fee Act (PDUFA), which was first created by Congress in 1992 to authorize the FDA to collect fees from companies to expedite the drug approval process and reauthorized every 5 years, has performance goals for the review timelines to be 10 months from filing dates of both standard NDAs and BLA* ***[113]****.* | **21 CFR Part 314.100 +**  **21 USC 355c**  **21 CFR Part 314.110**  **21 CFR Part 601 +**  **42 USC 262**  *21 USC 379g to 379h-2* |
|  | **Clinical Trials Requirements** | Applications for new molecular entities and original BLAs require full set of clinical efficacy and safety reports as per the standard pathway.  The design of the main clinical trials supporting the application should be controlled and adequate measures to be taken to minimize bias and assure comparability such as randomization and blinding.  Uncontrolled or partially controlled studies can be supportive. However, they cannot be accepted as sole basis for approval of effectiveness claims.  The number of required substantial or pivotal clinical trials is not specified by regulation. | **21 USC 355 (b)(1)**  **21 CFR 314.126** |
|  | **Post-Authorization Studies** | The secretary shall request post-marketing studies upon agreement with the sponsor, which should be submitted within 1 year after the approval of the drug and annually tracked thereafter until the study is completed or terminated.  The post marketing studies, or clinical trials can be requested at the time of approval or after approval if the Secretary becomes aware of new safety information. | **21 USC 356b**  **21 USC 355(o)** |
| **New Indication MAs of already Authorized Entities** | **Procedure** | For post changes to an approved NDA or BLA, there are three types of changes:  - Major changes which require supplement submission and prior approval before distribution. For these changes, expedited review can be requested for public health reasons.  - Moderate changes which require supplement submission at least 30 days before distribution.  - Minor Changes which can be described in annual report.  In standard cases, a new therapeutic indication addition to already authorized drug or biological is considered a major change and needs supplemental application submission with the required evidence and prior approval by the FDA. | **21 CFR 314.70**  **21 CFR 601.12** |
|  | **Approval Timelines** | Review timelines for supplemental applications are not specified by related regulations.  *Note: The Prescription Drug User Fee Act (PDUFA) has performance goals for supplemental efficacy and manufacturing changes as well to be 10 months from filing date of standard applications and 6 months for priority review applications* ***[113]****.* | **21 CFR 314.70**  **21 CFR 601.12**  *21 USC 379g to 379h-2* |
| **Facilitated Pathways or Tools used for Covid-19 MAs** | **Emergency Use Authorization** | **Criteria and Conditions:**  EUA is applicable for drugs, biologics, and devices.  The FDA issues the EUA subject to conditions based on following criteria:  1) if the medical product is for serious or life-threatening disease or condition,  2) if there is evidence of effectiveness,  3) if benefits outweigh the potential known risks,  4) if there are no alternatives.  **Procedure**:  When the Secretary of the Department of Health and Human Services HHS declares that that the emergency use authorization is appropriate, the FDA may authorize unapproved medicinal product or unapproved use of approved medicinal product for the emergency cases following EUA application by the sponsor. This declaration is based on one of the four types of determinations for threats or potential threats issued by the Secretary of HHS, Homeland Security, or Defense.  **Approval Timeline:**  The EUA application review timeline by the FDA is not specified by the regulation. However, the FDA shall take actions to expedite the development and review of medicinal products for emergency uses.  *Note: FDA is prepared to issue EUA expeditiously (i.e., within hours or days) according to a guidance published by US FDA* ***[7]****.*  **Validity:**  The EUA will stay effective until termination or revocation of the declaration by secretary when the circumstances for issuance of such authorization are no longer exist. | **21 USC 360bbb-3**  **21 USC 360bbb-3c** |
|  | **Fast Track Designation** | **Criteria & Conditions:**  The designation shall facilitate the development and expedite the review of the drug application if it is intended for a serious or life-threatening disease or condition treatment and if it addresses unmet medical needs, or if the Secretary designates the drug as a qualified infectious disease product under US Code section 355f(d).  **Procedure**:  Fast track designation can be requested by the sponsor with or any time after submission of an application for the investigation of the drug. The FDA shall response to the request within 60 calendar days from the receipt of the request.  The regulation does not specify rolling review procedure associated with this designation.  *Note: A guideline published by FDA for expedited programs mentions additional rolling review feature for fast-track designation* ***[6]****.*  **Approval Timeline:**  The related regulation does not specify any review timeline.  **Validity:**  NA | **21 USC 356(b)** |
|  | **Priority Review** | **Criteria & Conditions:**  Priority review voucher can be granted  - If the drug is for serious condition and provides significant improvement in safety or effectiveness.  - If the drug is designated as a qualified infectious disease product.  - If the drug is intended for rare pediatric disease.  - If the drug is for treatment of agents that present national security threats.  **Procedure:**  Priority Review voucher can be issued by the Secretary to the sponsor upon request (with original NDA, BLA or efficacy supplement).  **Approval Timeline:**  The priority review voucher shall ensure the review and action by the Secretary not later than 6 months after receipt by the Secretary of such application.  It is referenced also to Prescription Drug User Fee Act, which has performance goals for the review timelines to be 6 months from filing date for priority review NDAs, BLAs or supplemental changes applications **[113].**  **Validity:**  NA | **21 USC 360bbb-4a**  **21 USC 360n-1**  **21 USC 360ff**  **21 USC 379g to 379h-2** |

# **FIGURES**

Figure 1: Comparison between EMA and USFDA in Number and Type of Covid-19 MAs
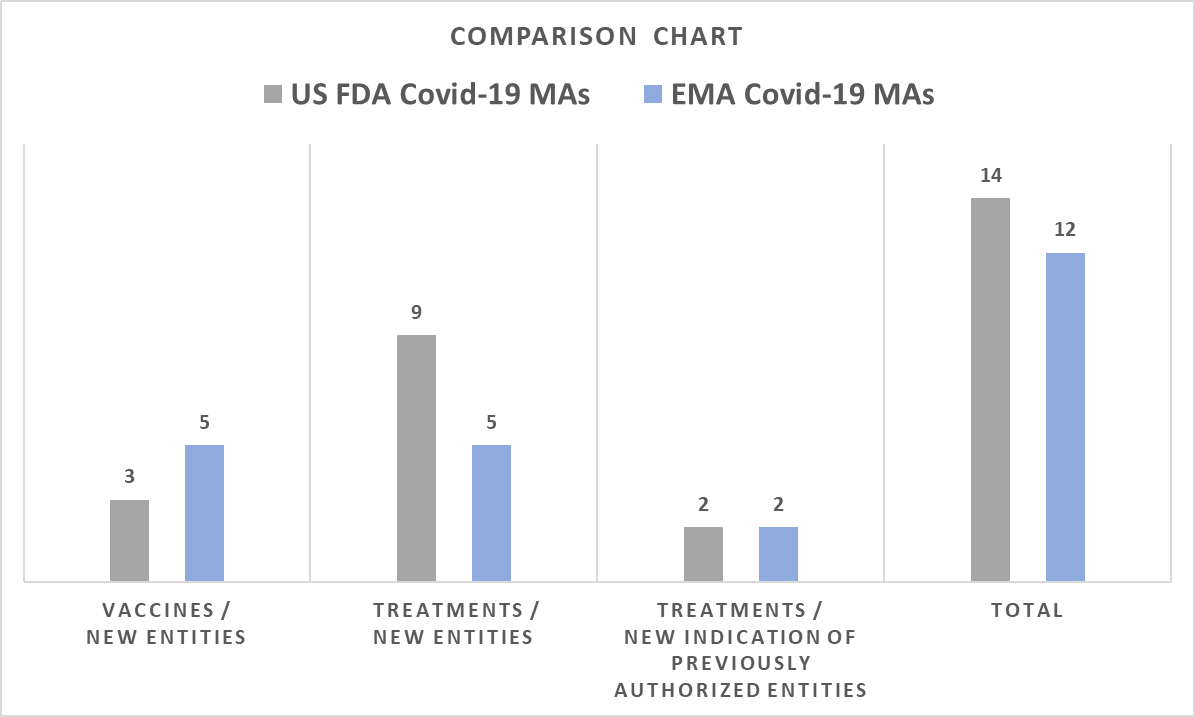


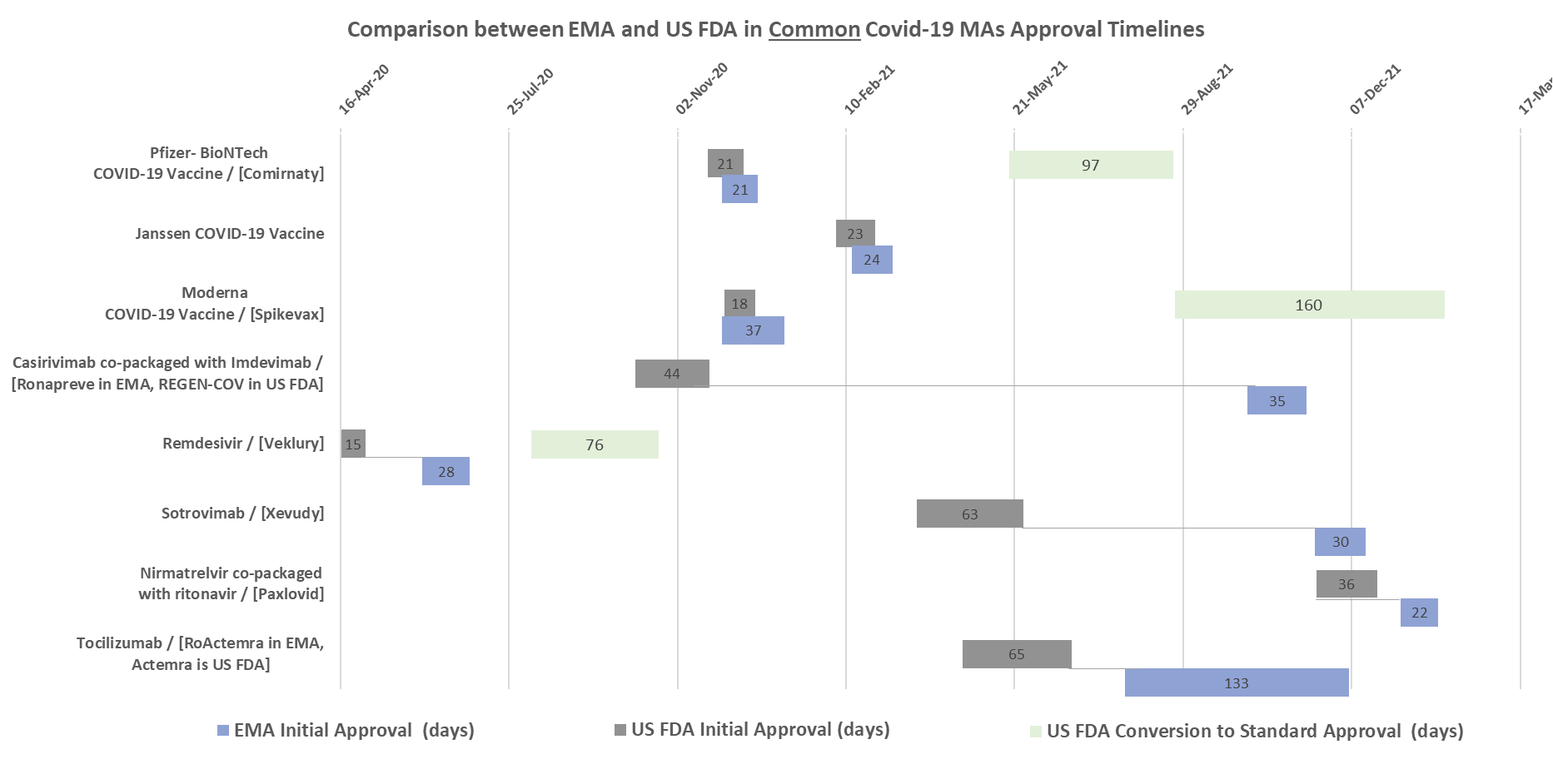
Figure 2: Comparison between EMA and USFDA in Approval Timelines for Covid-19 MAs Authorized by Both Authorities


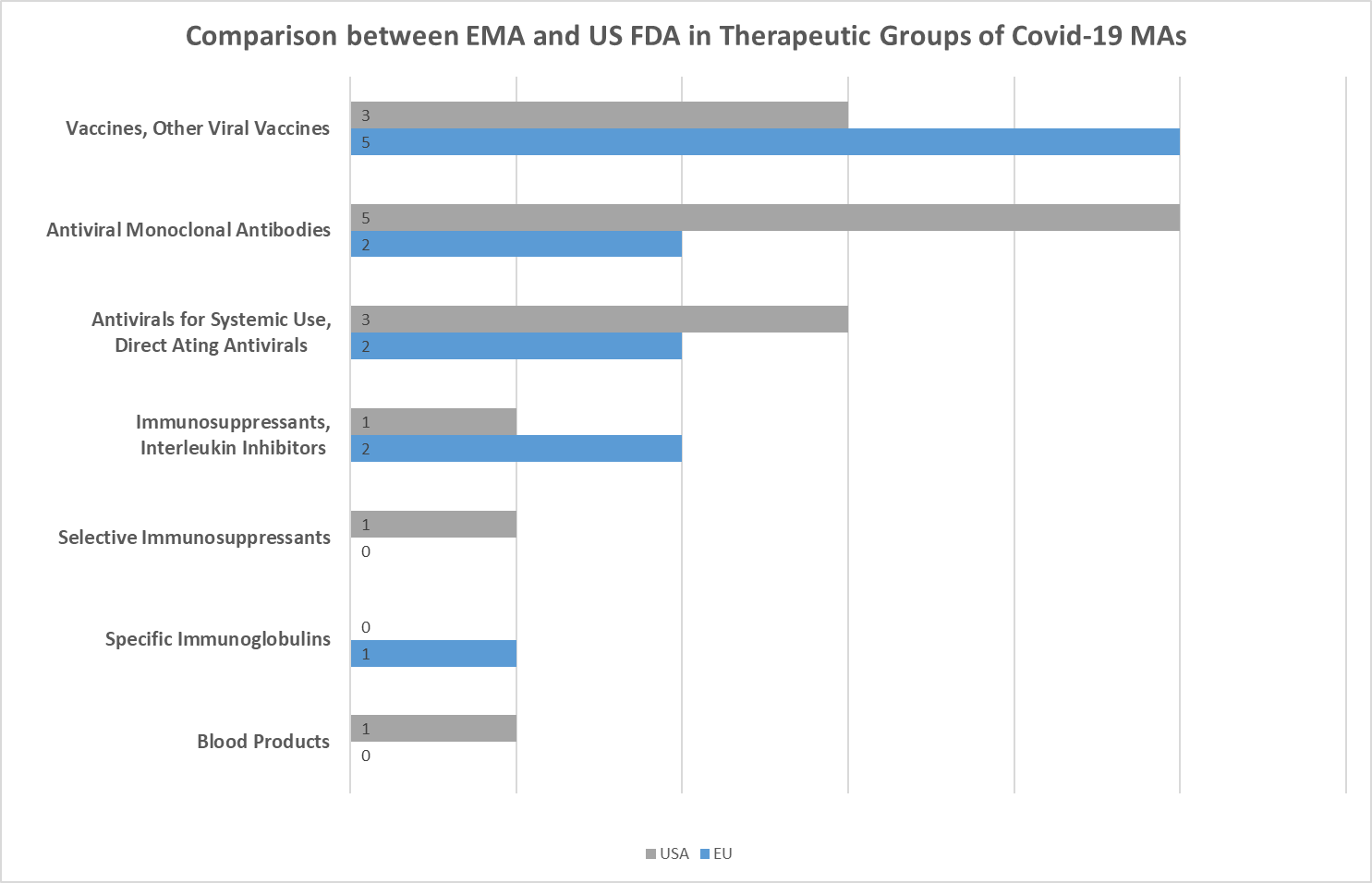
Figure 3: Comparison between Therapeutic Groups of USFDA and EMA Covid-19 MAs


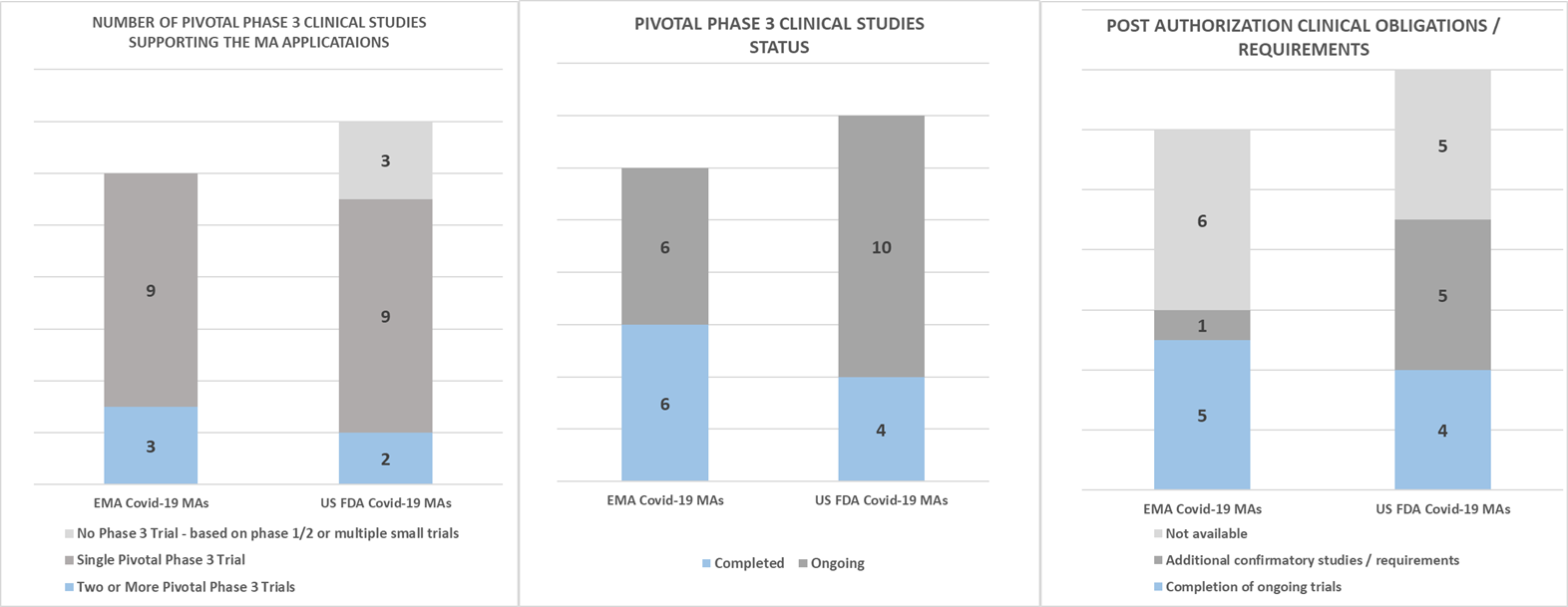
Figure 4: Comparison between EMA and USFDA in the Accepted Number / Status of Pivotal Clinical Trials and Post-Authorization Clinical Obligations

Figure 5: EU MA Centralized Procedure both for Standard and Implemented Covid-19 Cases


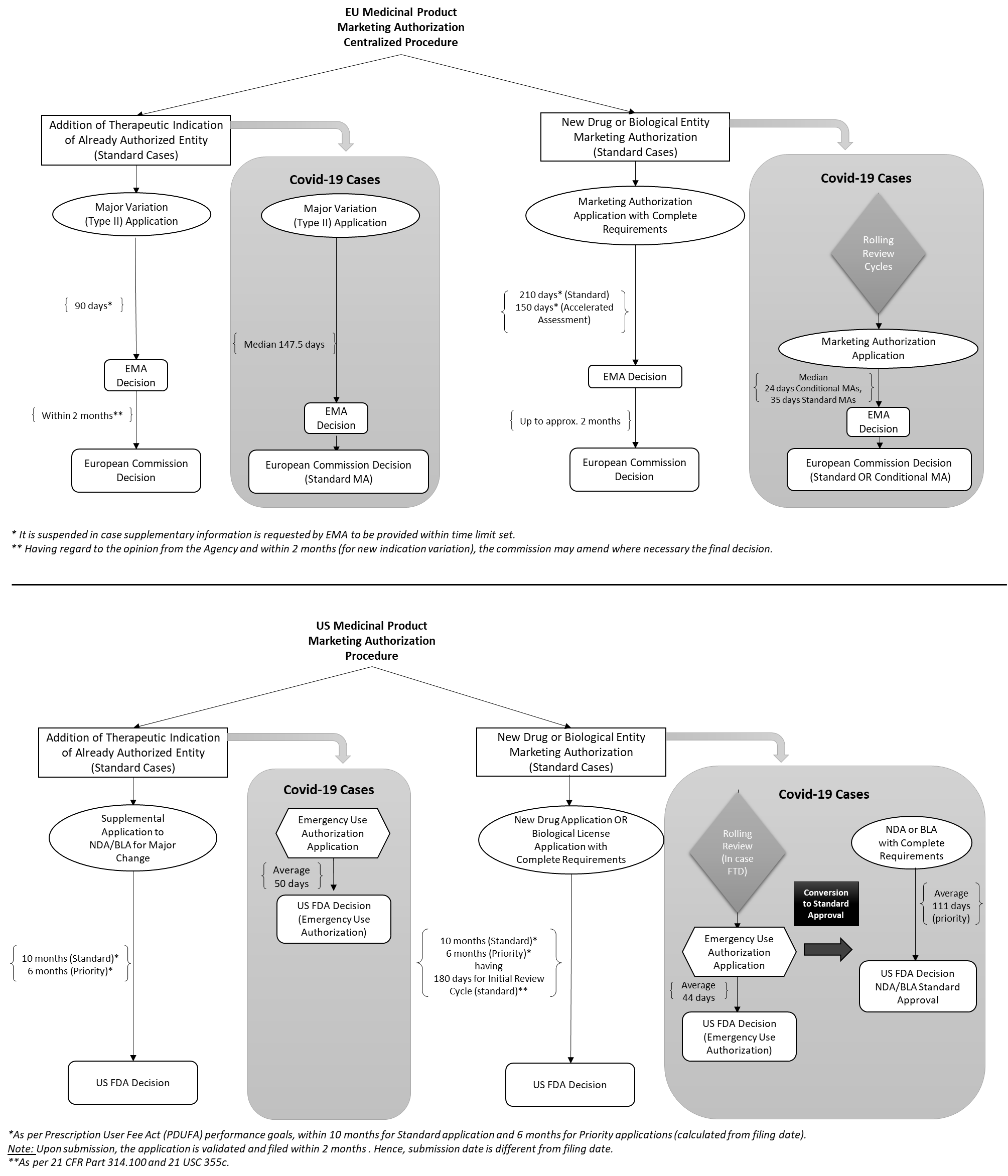


Figure 6: US Procedures both for Standard and Implemented Covid-19 Cases


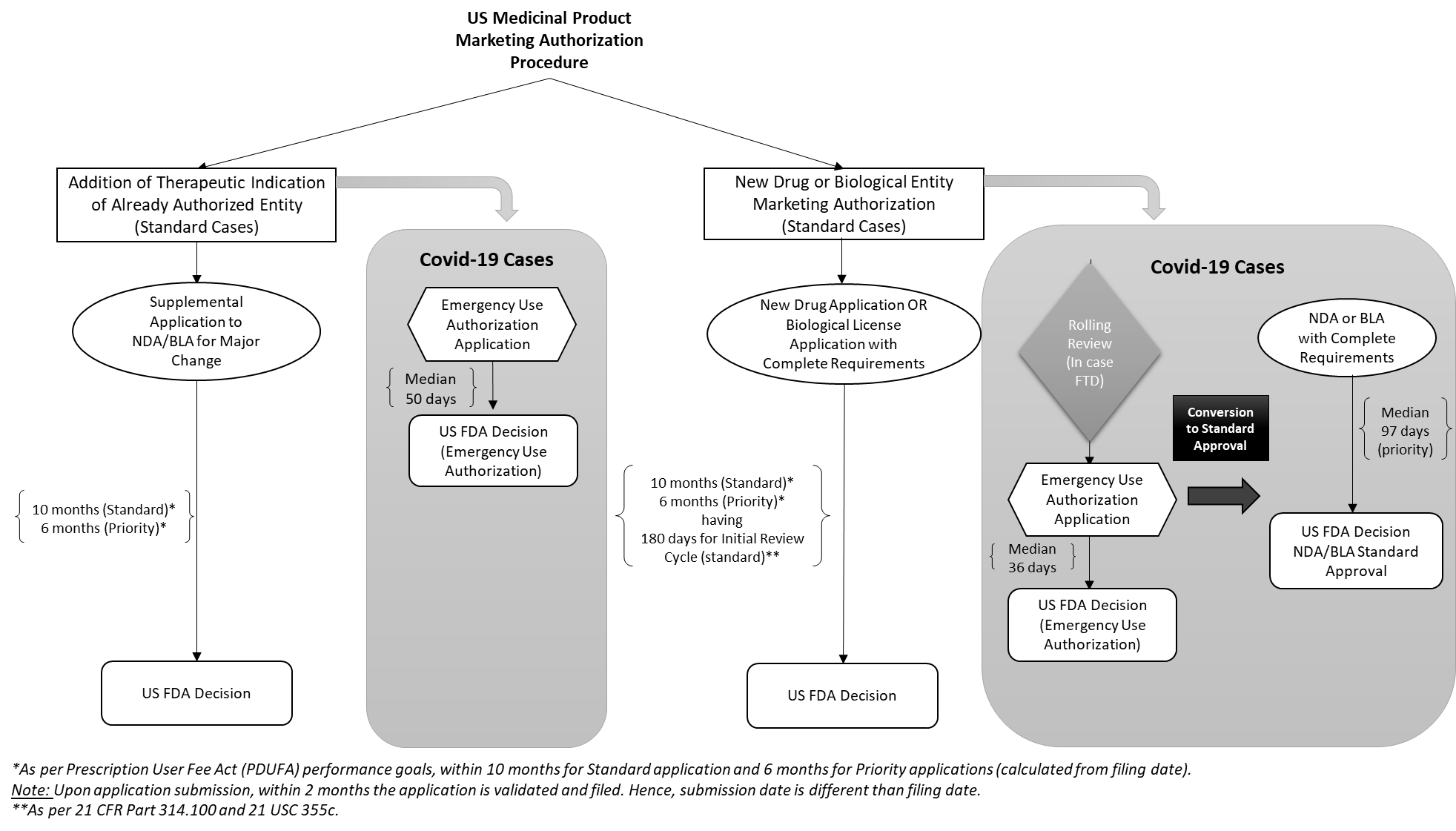


# **APPENDIX 1 - Definitions**

## **US FDA Definitions**

The following are the definitions of some of the regulatory US terms, which are relevant to this work. They are extracted from US FDA glossary of available terms **[114]**:

**Active Ingredient:** An active ingredient is any component that provides pharmacological activity or other direct effect in the diagnosis, cure, mitigation, treatment, or prevention of disease, or to affect the structure or any function of the body of man or animals.

**Approval Letter:** An official communication from FDA to a new drug application (NDA) sponsor that allows the commercial marketing of the product.

**Biologic License Application (BLA)****:** Biological products are approved for marketing under the provisions of the Public Health Service (PHS) Act. The Act requires a firm who manufactures a biologic for sale in interstate commerce to hold a license for the product. A biologics license application is a submission that contains specific information on the manufacturing processes, chemistry, pharmacology, clinical pharmacology and the medical affects of the biologic product. If the information provided meets FDA requirements, the application is approved and a license is issued allowing the firm to market the product.

**Biological Product:** Biological products include a wide range of products such as vaccines, blood and blood components, allergenics, somatic cells, gene therapy, tissues, and recombinant therapeutic proteins. Biologics can be composed of sugars, proteins, or nucleic acids or complex combinations of these substances, or may be living entities such as cells and tissues. Biologics are isolated from a variety of natural sources — human, animal, or microorganism — and may be produced by biotechnology methods and other cutting-edge technologies. Gene-based and cellular biologics, for example, often are at the forefront of biomedical research, and may be used to treat a variety of medical conditions for which no other treatments are available.

**Company****:** The company (also called applicant or sponsor) submits an application to FDA for approval to market a drug product in the United States.

**Drug**: A drug is defined as:

- A substance recognized by an official pharmacopoeia or formulary.
- A substance intended for use in the diagnosis, cure, mitigation, treatment, or prevention of disease.
- A substance (other than food) intended to affect the structure or any function of the body.
- A substance intended for use as a component of a medicine but not a device or a component, part or accessory of a device.
- Biological products are included within this definition and are generally covered by the same laws and regulations, but differences exist regarding their manufacturing processes (chemical process versus biological process.)

**Drug Product****:** The finished dosage form that contains a drug substance, generally, but not necessarily in association with other active or inactive ingredients.

**Generic Drug****:** A generic drug is the same as a brand name drug in dosage, safety, strength, how it is taken, quality, performance, and intended use. Before approving a generic drug product, FDA requires many rigorous tests and procedures to assure that the generic drug can be substituted for the brand name drug. The FDA bases evaluations of substitutability, or "[therapeutic equivalence](https://www.fda.gov/drugs/drug-approvals-and-databases/drugsfda-glossary-terms#TE)," of generic drugs on scientific evaluations. By law, a generic drug product must contain the identical amounts of the same active ingredient(s) as the brand name product. Drug products evaluated as "therapeutically equivalent" can be expected to have equal effect and no difference when substituted for the brand name product.

**New Drug Application (NDA)****:** When the sponsor of a new drug believes that enough evidence on the drug's safety and effectiveness has been obtained to meet FDA's requirements for marketing approval, the sponsor submits to FDA a new drug application (NDA). The application must contain data from specific technical viewpoints for review, including chemistry, pharmacology, medical, biopharmaceutics, and statistics. If the NDA is approved, the product may be marketed in the United States. For internal tracking purposes, all NDAs are assigned an NDA number.

**New Molecular Entity (NME)****:** An NME is an active ingredient that contains no active moiety that has been previously approved by the Agency in an application submitted under section 505 of the Federal Food, Drug, and Cosmetic Act, or has been previously marketed as a drug in the United States**.**

**Review****:** A review is the basis of FDA's decision to approve an application. It is a comprehensive analysis of clinical trial data and other information prepared by FDA drug application reviewers. A review is divided into sections on medical analysis, chemistry, clinical pharmacology, biopharmaceutics, pharmacology, statistics, and microbiology.

**Supplement****:** A supplement is an application to allow a company to make changes in a product that already has an approved new drug application (NDA). CDER must approve all important NDA changes (in packaging or ingredients, for instance) to ensure the conditions originally set for the product are still met.

## **EMA Definitions**

The following are the definitions of some of the regulatory EU terms, which are relevant to this work. They are extracted from EMA glossary of available terms **[115]**:

**Active substance:** The substance responsible for the activity of a medicine.

**Advanced therapy medicinal product:** A medicine for human use that is based on genes, cells or tissue engineering.

**ATC code:** The Anatomical Therapeutic Chemical code: a unique code assigned to a medicine according to the organ or system it works on and how it works. The classification system is maintained by the World Health Organization (WHO).

**Biological medicine:** A medicine whose active substance is made by a living organism.

**Biosimilar medicine:** A medicine that is similar to a biological medicine that has already been authorised.

**Centralised procedure:** The European Union-wide procedure for the authorisation of medicines, where there is a single application, a single evaluation and a single authorisation throughout the European Union. Only certain medicines are eligible for the centralised procedure.

**Centrally authorised product:** A medicine with a single marketing authorisation issued by the European Commission and valid across the European Union.

**CHMP:** Committee for Medicinal Products for Human Use: the committee that is responsible for preparing the Agency's opinions on questions concerning human medicines.

**Clinical trial:** A study performed to investigate the safety or efficacy of a medicine. For human medicines, these studies are carried out in human volunteers

**Committee for Medicinal Products for Human Use:** The committee that is responsible for preparing the Agency's opinions on questions concerning human medicines. Abbreviated as CHMP.

**Conditional marketing authorization:** The approval of a medicine that addresses unmet medical needs of patients on the basis of less comprehensive data than normally required. The available data must indicate that the medicine’s benefits outweigh its risks and the applicant should be in a position to provide the comprehensive clinical data in the future.

**Efficacy:** The measurement of a medicine's desired effect under ideal conditions, such as in a clinical trial.

**European Commission decision:** The legally binding decision issued by the European Commission at the end of a regulatory procedure, such as a marketing authorisation application or arbitration procedure. A European Commission decision comes after an opinion from one of the Agency’s scientific committees.

**European public assessment report:** A set of documents describing the evaluation of a medicine authorised via the centralised procedure and including the product information, published on the European Medicines Agency website. European public assessment reports include the product information. Abbreviated as EPAR.

**Generic medicine:** A generic medicine is a medicine that is developed to be the same as a medicine that has already been authorised. Its authorisation is based on efficacy and safety data from studies on the authorised medicine. A company can only market a generic medicine once the 10-year exclusivity period for the original medicine has expired.

**Guideline:**A document providing guidance on the scientific or regulatory aspects of the development of medicines and applications for marketing authorisation. Although guidelines are not legally binding, applicants need to provide justification for any deviations.

**Indication:** A medical condition that a medicine is used for. This can include the treatment, prevention and diagnosis of a disease.

**Innovative medicine:** A medicine that contains an active substance or combination of active substances that has not been authorised before.

**Investigational medicinal product:** A medicine being studied in a clinical trial.

**Marketing authorization:** The approval to market a medicine in one, several or all European Union Member States.

**Marketing authorisation application:** An application made to a European regulatory authority for approval to market a medicine within the European Union.

**Marketing authorisation holder:** The company or other legal entity that has the authorisation to market a medicine in one, several or all European Union Member States.

**Medicinal product:** A substance or combination of substances that is intended to treat, prevent or diagnose a disease, or to restore, correct or modify physiological functions by exerting a pharmacological, immunological or metabolic action.

**Off-label use:** Use of a medicine for an unapproved indication or in an unapproved age group, dosage, or route of administration.

**Pharmaceutical form:** The way a medicine is presented, e.g. tablet, capsule, solution for injection, cream, etc.

**Phase-I study:** A type of clinical study where a new medicine is given to humans for the first time, usually in healthy volunteers. It looks at the way the medicine is dealt with by the body, its main effects and main side effects.

**Phase-II study:** A type of clinical study conducted after phase I studies to evaluate a medicine’s effects in a particular condition and to determine its common short-term side effects.

**Phase-III study:** A type of clinical study usually conducted in a large group of patients to gather information about a medicine's efficacy and safety, to allow its benefits and risks to be evaluated.

**Phase-IV study:** A type of clinical study that takes place after the authorisation of a medicine.

**Regulatory authority:** A body that carries out regulatory activities relating to medicines, including the processing of marketing authorisations, the monitoring of side effects, inspections, quality testing and monitoring the use of medicines.

**Renewal:** An extension of the validity of a marketing authorisation, which can be for a fixed or indefinite period of time. Initial marketing authorisations are usually valid for five years.

**Specific obligations:** Requirements imposed on holders of conditional marketing authorisations or marketing authorisations granted under exceptional circumstances.

**Type IA variation:** A minor change to a marketing authorisation that has a minimal or no impact on the quality, safety or efficacy of the medicine and does not require prior approval before implementation by the marketing authorisation holder.

**Type IB variation:** A minor change to a marketing authorisation that the marketing-authorisation holder must notify to the regulatory authority before implementation, but which does not require formal approval.

**Type II variation:** A major change to a marketing authorisation that may have a significant impact on the quality, safety or efficacy of a medicine, but does not involve a change to the active substance, its strength or the route of administration. Type II variations require a formal approval.
